# Electrophysiological signatures of ongoing thoughts during naturalistic behavior

**DOI:** 10.1162/IMAG.a.20

**Published:** 2025-06-05

**Authors:** Julia W.Y. Kam, Tarannum Rahnuma, Sairamya Nanjappan Jothiraj, Alexandra A. Ouellette-Zuk, Robert T. Knight

**Affiliations:** Department of Psychology, University of Calgary, Calgary, Canada; Hotchkiss Brain Institute, University of Calgary, Calgary, Canada; Department of Social Work, University of Toronto, Toronto, ON, Canada; Helen Wills Neuroscience Institute, Department of Psychology, University of California, Berkeley, Berkeley, CA, United States

**Keywords:** ongoing thoughts, mind wandering, spontaneous thought, EEG, naturalistic tasks, deep learning

## Abstract

Humans engage in a continuous stream of ongoing mental experience. Recent work examining the neural correlates of several dimensions of thoughts has revealed their functional connectivity patterns using fMRI during constrained experimental tasks. Less is known about the electrophysiological basis of various thoughts dimensions in more naturalistic settings. To address this, we first examined the electrophysiological signatures of ongoing thoughts during naturalistic tasks in seven participants across seven recording sessions. We then combined deep learning algorithms with electrophysiological data to determine the utility of these signals in predicting thought dimensions. Based on a total of 49 data sets, our results revealed distinct oscillatory markers of 7 dimensions of ongoing thought as participants completed any computer-based activities they wished to perform. In addition to identifying electrophysiological markers consistent with those observed in experimental settings for internally oriented thoughts and freely moving thoughts, we found novel patterns not previously reported for off-task thoughts, goal-oriented thoughts, and sticky thoughts, primarily characterized by spectral activity in canonical theta, alpha, and beta bands. Importantly, applying deep learning algorithms on electrophysiological data reliably detected all seven thought dimensions at above chance levels for both within-participant (MCC = 0.22–0.43) and across-participant (MCC = 0.14–0.31) approaches. Together, these results established the electrophysiological signatures of seven dimensions of ongoing thought, assembling a comprehensive set of brain-to-experience mapping of the phenomenological features of thoughts. Our findings provide an important step toward predicting thought patterns in the real world with clinical implications for establishing biomarkers of typical and atypical thought patterns.

## 1 Introduction

As William James aptly described over a century ago, our ongoing inner experience is characterized by an uninterrupted stream of thoughts. Thus far, neuroimaging research has mainly focused on the nature of our thoughts during highly controlled experimental tasks, leaving our ongoing thoughts during naturalistic behavior remaining largely unexplored. In addition to being a core feature of the human experience, the importance of understanding the nature of our ongoing thoughts also stems from its close association with affective states (Kam et al., 2024) and clinical conditions (Kucyi et al., 2023). While recent work has begun to explore various dimensions of ongoing thoughts in the laboratory and their corresponding functional connectivity patterns, little is known about their electrophysiological basis. The current study examined the electrophysiological signatures of seven dimensions of our ongoing thoughts during naturalistic behavior across multiple recording sessions.

Earlier studies have primarily focused on a single thought dimension, with most research examining thoughts that are unrelated to the task at hand. While these off-task thoughts have important theoretical implications (Smallwood & Schooler, 2015), real-world applications (Dong et al., 2021;Jin et al., 2019;Mills et al., 2021) as well as clinical implications (Arch et al., 2021;Hoffmann et al., 2016;Kucyi et al., 2023), it leaves out other important characteristics about our thoughts. As an example, it does not capture the*dynamics*of our thoughts (Christoff et al., 2016;Irving, 2016). The dynamic framework of spontaneous thought highlights the different ways in which our ongoing thoughts unfold over time. When unconstrained, our thoughts that freely move from one topic to another have been associated with positive affect (Mills et al., 2018;Thiemann et al., 2023). In contrast, our thoughts can also be constrained to a particular topic: either being automatically drawn with minimal control to critical events in our life (i.e., a defining feature of rumination and a core symptom of depression) or deliberately toward our goals requiring cognitive control. Another example concerns the*content*of our thoughts: simply knowing one is not focusing on the task does not inform where their thoughts are focused. A commonly reported set of content-related thought dimensions are thoughts about ourselves and others, which have contrasting affective outcomes (seeKam et al., 2024for a review). For example, thinking about oneself in the future has been linked to positive affect, whereas thinking about others in the past has been linked to negative affect (Ruby et al., 2013). These separate lines of research demonstrate the variety of dimensions that characterize our ongoing thoughts with unique corresponding implications. Therefore, examining a range of dimensions is crucial for developing a comprehensive picture of the content and dynamics of our ongoing thoughts.

There has been a surge of interest in simultaneously examining multiple dimensions of our ongoing thoughts using the multi-dimensional experience sampling (MDES) approach (Mckeown et al., 2021;Smallwood et al., 2021;Turnbull et al., 2019;H.-T. Wang et al., 2020). MDES involves sampling a number of dimensions of one’s ongoing mental experience at any given moment in time, and has been mainly applied to functional neuroimaging data (Konu et al., 2020;Turnbull et al., 2019). These fMRI studies documented brain activity and connectivity patterns associated with thought dimensions but lack the sub-second temporal resolution to reveal neurophysiological underpinnings of thought. Electroencephalogram (EEG) offers the temporal resolution necessary to examine spectral activity linked to cognitive function (Siegel et al., 2012;Ward, 2003) and is amenable to practical applications, including real-time predictions of thought dimensions in a naturalistic setting (Kam et al., 2019). To date, one study has examined electrophysiological data in combination with MDES and focused on the P300 event-related potential (ERP) component (Simola et al., 2023). Our systematic review has reported opposing patterns between ERP and spectral markers of off-task thoughts, suggesting these two types of markers may capture different information about the underlying cognitive process (Kam et al., 2022). Notably, although the spectral signatures of the off-task thought dimension have been established (Kam et al., 2022), little is known about the signatures of other thought dimensions. This includes dimensions assessed in the current study pertaining to attentional focus of thought (i.e., internal or external), thought dynamics (i.e., thoughts that are freely moving from one topic to another, goal-oriented, or stuck on a topic), and thought content (i.e., thoughts about the self and others).

Most studies of ongoing thoughts have examined neural signatures during experimental tasks characterized by strictly controlled paradigms. While evidence suggests that some thought dimensions emerge within the laboratory and in the real world (Kane et al., 2017;Kuehner et al., 2017;McVay et al., 2009), it is less known whether the electrophysiological signatures of those thought dimensions are similarly consistent across experimental and naturalistic tasks. The few studies that have examined specific thought dimensions in a naturalistic setting (Dhindsa et al., 2019) or used simulations of real-world tasks in a laboratory (Boudewyn & Carter, 2018;Conrad & Newman, 2021;Tang et al., 2023) have identified electrophysiological markers of off-task thoughts, which generally align with those reported during experimental tasks (Kam et al., 2022). Similarly, studies that have combined machine or deep learning approaches with electrophysiological data have mainly focused on detecting off-task thoughts. These studies have reported several electrophysiological measures, including the P3 ERP component and alpha activity, which successfully predicted off-task thought occurrence in experimental (Dong et al., 2021;Groot et al., 2021;Jin et al., 2019,^2023^) and naturalistic settings (Dhindsa et al., 2019). A recent study also found that machine learning applied on these EEG measures can detect freely moving thought with above chance accuracy (Jothiraj et al., 2025). What remains unknown are the electrophysiological markers of other thought dimensions during naturalistic tasks or behavior, and whether they can be combined with deep learning to detect the occurrence of thought dimensions.

An important consideration concerning the experience sampling approach is the limited amount of data typically included in analysis. There are two reasons for this. First, since this approach places no restrictions on the types of thoughts that participants experience, they may report little or no occurrence of any given thought dimension in a single recording session. Although past studies have consistently reported the frequency of off-task thoughts to range from 25% to 50% (Smallwood & Schooler, 2015), the occurrence of other thought dimensions has not been well established. Second, the experience sampling approach typically restricts the analyses of EEG data to a certain time interval (around 12 seconds) preceding the thought probe that elicits the report (Dhindsa et al., 2019;Kam et al., 2021;Polychroni et al., 2022), based on the assumption that these reports represent their thoughts occurring within the preceding time interval (Compton et al., 2019;O’Connell et al., 2009). Accordingly, studies may run into the risk of not obtaining sufficient data within a single session to obtain a reliable EEG measure of a given thought dimension. From that perspective, implementing multiple recording sessions allows for the acquisition of a large amount of data for each participant to achieve a more reliable estimate of thought content (Hung & Hsieh, 2022;Kucyi et al., 2024;Poldrack et al., 2015). Similar to these studies, we prioritized acquiring a large amount of data from a smaller sample in order to obtain sufficient data for each thought dimension.

The current study, therefore, examined the electrophysiological basis of dimensions of our ongoing thoughts during naturalistic tasks in seven participants across seven recording sessions. At each session, seven participants were asked to complete any tasks they wish to perform on a computer and occasionally report the nature of their ongoing thoughts, including task relatedness, attentional focus of thought (i.e., internal or external), thought dynamics (i.e., thoughts that are freely moving, goal oriented, or stuck on a topic), and thought content (i.e., thoughts about the self and others). In order to assess whether these signatures were observed without the influence of dynamically changing external stimuli, participants were also asked to retrospectively report their thoughts during two resting-state recordings. First, we used a data-driven approach to establish distinct electrophysiological profiles of each of the seven thought dimensions during naturalistic tasks and then assessed their electrophysiological markers during rest. We then examined the similarities and differences in these signatures of thought dimensions between the tasks and rest conditions. Finally, we combined deep learning algorithms with electrophysiological data to determine the utility of these signals in predicting thought dimensions. Together, this study aims to establish the electrophysiological basis of seven dimensions of ongoing thought using several data-driven approaches.

## 2 Methods

### 2.1 Participants

Seven individuals (4 females and 3 males; age: M = 24.57 years, S.D. = 3.66; years of education: M = 16.4, S.D. = 1.51) participated in the study. They were undergraduate and graduate students primarily recruited from the Department of Psychology at the University of Calgary. All participants had normal or corrected-to-normal vision and did not self-report neurological disorders. They provided written informed consent and were paid for their participation. The study was approved by the Conjoint Faculty Review Ethics Board at the University of Calgary.

### 2.2 Experimental procedure

Participants completed seven recording sessions, with each session lasting approximately 2 hours. They were first asked to complete an eyes-open resting-state recording. Following this, they were asked to complete any tasks they wish to perform. During this time, they were occasionally presented with thought probes that prompted them to report the nature of their ongoing thoughts (which we describe below). Finally, they completed a second round of eyes-open resting state. We recorded EEG data during each of these components.

### 2.3 Self-selected tasks and thought probes

During each session, participants were asked to complete any task they wished to perform on the provided computer for approximately 80 minutes. This freedom to choose their own activities was designed to engage them in naturalistic behavior and enhance the ecological validity of the “task.” Given that many jobs require individuals to work on a computer and remote work has become more prevalent, we considered their self-selected tasks to mimic their everyday activity. Based on participants’ reports of their self-selected task (as described below), these tasks were coded into the following categories: reading, writing/editing, watching videos, browsing or surfing the internet, other more cognitively demanding tasks (e.g., games such as sudoku, creating PowerPoint presentations, programming), and others (e.g., doing nothing). As participants were free to select any activities (or no activities), the response “nothing” to the open-ended question of what activity they were performing indicates that participants were not performing any activities at that time.

Throughout the self-selected tasks, multi-dimensional experience sampling (MDES) thought probes occurred approximately every 120 seconds (range: 90–150 seconds to minimize expectation effects). These probes were presented in a pop-up dialogue box displaying a short list of questions, along with a tone (1000 Hz, 200 m seconds) to alert participants of its occurrence. At each thought probe, participants answered several questions about their ongoing experience immediately preceding the probe. They were first asked to briefly report their self-selected task (in a few words), defined as the activity they were currently doing. Following this, we asked them to describe their thoughts in terms of form and content by responding to questions on a 7-point Likert scale outlined inTable 1. These represent some of the most commonly examined thought dimensions in the literature within the context of a task (Mills et al., 2018;Ruby et al., 2013;Simola et al., 2023;Wang et al., 2020). Participants were asked to rate on two additional dimensions related to thought modality, visual and auditory, which were included for exploratory purposes. Further information about these two dimensions is reported in theSupplementary Materials. To ensure that participants understood the meaning of these questions, we provided definitions and example scenarios of each question prior to beginning the task (seeSupplementary Table S1). There was a total of 35 thought probes presented during each session. This self-selected task format allowed us to assess the oscillatory markers of thought dimensions regardless of the specific task participants chose to be engaged in.

**Table 1.** Multi-dimensional experience sampling thought probe questions.

Questions	Response scale
What were you doing?	Open-ended response
To what extent were your thoughts on-task vs. off-task?	On-task to off-task
To what extent were your thoughts freely moving?	Not at all to very much
To what extent did you have difficulty disengaging from your thoughts?	Not at all to very much
To what extent were your thoughts goal directed on a topic?	Not at all to very much
To what extent were your thoughts focused on your inner thoughts vs. external stimuli?	Internal to external
To what extent were your thoughts about yourself?	Not at all to very much
To what extent were your thoughts about another person/other people?	Not at all to very much

*Note*: All questions except for the first were responded on a 7-point Likert scale. These questions were asked during naturalistic tasks and were identical in content as those asked during resting state, with the exception that the first two questions were only asked during the task condition and not during the resting-state condition.

### 2.4 Resting-state and post-rest thought probe

For the two resting-state recordings within each session, participants were asked to keep their eyes open and fixated on a central fixation cross for 5 minutes. Following this, they were presented with a series of questions that asked them to retrospectively report their overall thoughts during those 5 minutes. Since the resting-state recordings only lasted 5 minutes, we did not implement the experience sampling approach used during task as this would lead to an insufficient number of trials required for subsequent analysis. Rather, we implemented a retrospective approach that captured the overall levels of thoughts across the entire 5-minute recording.

The time interval of interest captured using these questions contrasts with the experience sampling approach during self-selected tasks, which asked about their ongoing thoughts in the moment. Notably, the content of these questions was identical to the questions asked at each thought probe during the task (as reported inTable 1). The only exception was that participants were not asked about their task or the task relatedness of their thought, as these questions were not relevant to the resting state. This resting-state condition allowed us to assess the oscillatory markers of thought dimensions without the influence of changes in external stimuli.

### 2.5 EEG data acquisition and preprocessing

The following data acquisition, preprocessing, and quantification steps were implemented for data recorded during the task and resting states. EEG was recorded continuously from a 32-channel wireless semi-dry Bitbrain Versatile EEG system mounted on a cap according to the 10–20 layout. Vertical and horizontal eye movements were recorded from electrodes above and below the right eye, and two electrodes placed at the right and left outer canthi. Data were amplified and digitized at a sampling rate of 256 Hz.

EEG preprocessing, quantification, and analyses were performed with the FieldTrip toolbox (Oostenveld et al., 2011) within MATLAB 2020b for preprocessing and analyses and MATLAB 2023b for quantification (The MathWorks Inc, 2023). EEG data were first bandpass filtered between 1 Hz (which is ideal for independent component analysis described below (Klug & Gramann, 2021)) and 55 Hz (which is below the line noise at 60 Hz) using a two-pass Butterworth filter with a hamming window. The signal was notch filtered at 60 Hz as well as harmonics at 120 Hz. Continuous data were then inspected for jump and muscle artifacts (through automatic detection) as well as noisy channels and segments (through visual inspection), which were then removed prior to the next step. Independent component analysis was then performed using the FastICA algorithm, to identify and remove artifactual components, such as ocular and muscular artifacts, based on component power spectral density, time course, and topography. Each trial was then visually inspected to identify remaining noisy segments, which were removed from subsequent analyses. Electrodes with excessively noisy signals were also identified via visual inspection and replaced using interpolation from neighboring electrodes. Continuous EEG data were then segmented into non-overlapping 2 second epochs, with 1 second preceding and following the 2 seconds of interest for padding purposes during spectral decomposition. Finally, a common average reference was applied to the data prior to subsequent analyses. The total number of artifact-free trials included in subsequent analyses for each participant and session is reported inSupplementary Table S2.

### 2.6 Quantification of oscillatory activity

Next, we quantified the oscillatory activity of the artifact-free EEG epochs in both the self-selected task and resting-state data. Notably, traditional quantification methods of spectral activity often do not differentiate between the periodic (or oscillatory) and aperiodic (often referred to as background activity) component (Donoghue et al., 2020). As these traditional methods do not account for the presence of aperiodic activity, it is possible that observed conditional or group differences in spectral activity may not reflect genuine oscillatory differences and may instead capture changes in the aperiodic component (such as the offset or slope of the 1/f, as illustrated in detail inDonoghue et al. (2020)). Contemporary methods of quantifying neural oscillations more precisely capture the peaks of narrowband power above the background aperiodic component, which is purported to indicate the genuine presence of periodic, rhythmic activity (Donoghue et al., 2020;Wen & Liu, 2016). Studies to date have only implemented traditional methods of quantifying neural oscillations of thought dimensions. In contrast, our approach establishes whether the oscillatory component is truly implicated in different thought dimensions as it removes the background aperiodic component from the signal.

To quantify the oscillatory component, we first implemented spectral decomposition for each 2 second epoch, using fast Fourier transform with Welch’s method of 0.5 second overlapping windows for frequencies between 4 and 30 Hz at 1 Hz steps. This range spans several canonical frequency bands, which we refer to as low theta (4–5 Hz), high theta (6–7 Hz), low alpha (8–9 Hz), high alpha (10–13 Hz), low beta (14–20 Hz), and high beta (21–30 Hz). Next, in order to account for the aperiodic component, we first quantified aperiodic activity using the FOOOF (Fitting Oscillations and One Over F) method (Donoghue et al., 2020). This method fits a linear component over the spectral curve that represents the 1/f slope, and this 1/f component was then subtracted from the original spectrum. This resulted in an estimate of true oscillatory activity (as represented by peaks in power spectral density plots) that accounts for aperiodic activity at each frequency and electrode collapsing across time, which is appropriate for these data given the absence of experimental stimuli for time locking of oscillatory activity. Accordingly, our interpretations of the results are focused on the peaks observed in these plots. The resulting electrode by frequency matrix was then subjected to statistical analysis.

### 2.7 Statistical analyses

#### 2.7.1 Self-selected tasks

To examine the spectral signatures of each thought dimension during self-selected tasks, we first categorized EEG data based on participants’ responses to the thought probes. Specifically, their responses to each question were dichotomized into two groups separating responses on the lower end (1–3) and upper end (5–7) of the Likert scale and discarding the middle response (4). For instance, their response to the task-related question would be categorized as on-task (responses 1–3), off-task (responses 5–7), or discarded if they responded 4 on the Likert scale. This approach is based on a pragmatic consideration (which we elaborate in theSupplementary Materials) concerning the cluster-based permutation tests (as described below) and is consistent with previous studies that also used a 7-point Likert scale for experience sampling questions (Kane et al., 2017;Smith et al., 2018;Stawarczyk et al., 2011).

#### 2.7.2 Time window of analysis

We only categorized the EEG data within the 12 seconds preceding the probe into their corresponding groups (lower and upper end) for each thought dimension. This is a commonly used time interval that aims to maximize the number of epochs needed to create a reliable average while still maintaining a reasonable validity of the participants’ report and is consistent with previously used time windows for capturing mental states using experience sampling methods (Kam et al., 2022). To ascertain whether the 12-second time window was appropriate for this data set, we also implemented the identical cluster-based permutation tests on EEG data within the 8 and 16 seconds preceding the probe. The direction and significance of results for each thought dimension with these two time windows were the same as the 12-second period. There were two exceptions in which the results based on the 8 and 16 second time window became non-significant. We report the cluster-based permutation test results of these two other time windows in theSupplementary Materials.

#### 2.7.3 Cluster-based permutation test

We used the cluster-based permutation test to determine the oscillatory activity and its topographic distribution associated with a given thought dimension. For each thought dimension, we compared trials on the upper versus lower end of the dimension, which conceptually aligns with the presence of that thought dimension for responses on the upper end of the Likert scale (e.g., off-task thought or freely moving thought) and the opposite or absence of that thought dimension for lower end responses (e.g., on-task thought or not freely moving thought). We averaged spectral power across trials within sessions associated with the upper or lower end responses, resulting in two electrode x frequency matrices per thought dimension. Sessions in which there were no trials included on either the upper or lower end of the given thought dimension were excluded from subsequent analyses. For example, if there were no trials associated with freely moving thoughts, either due to no reports or no artifact-free data, then that session for that participant was not included in the freely moving thought analyses. The electrode x frequency matrices for each session and participant were collated and then subjected to a cluster-based permutation test to determine differences between the upper and lower end of a given thought dimension. There were up to 49 data sets (7 sessions x 7 participants) included in a cluster-based permutation test for each thought dimension. This is comparable with or exceeds the number of data sets reported in recent studies using EEG to examine different types of thoughts (Dhindsa et al., 2019;Jin et al., 2019;Kam et al., 2021;Polychroni et al., 2022;Simola et al., 2023).

The advantage of a cluster-based permutation test approach is that it does not presume the region or frequency in which differences may emerge. This data-driven approach is especially relevant for the current study, which is the first to examine the electrophysiological correlates of numerous thought dimensions during naturalistic tasks, and lacks guidance from past studies on the choice of regions or frequencies of interest.

#### 2.7.4 Linear mixed-effects models as control analyses

We implemented two sets of control analyses. First, since the cluster-based permutation test treats each data set as independent and does not account for data originating from the same participant across sessions, we implemented linear mixed effects analyses to statistically account for this participant-level variance (through the inclusion of a random effect). Each model consisted of fixed within-participant effects of thought dimension (upper vs. lower end) as well as random intercepts of participant and session nested within participant. The dependent variable was the session-level mean of the EEG datapoints within the significant electrode x frequency clusters that emerged from the cluster-based permutation test. As the cluster-based permutation test already established the significance of the electrophysiological differences, the aim of the linear mixed effects analyses was to additionally verify whether the results remained significant after controlling for participant-level variance.

Second, to account for the influence of other correlated thought dimensions, we implemented another set of linear mixed effects analyses that statistically controlled for the ratings of the three most highly correlated dimensions with the thought dimension of interest within participants. These correlations were computed based on the participants’ response on the 7-point Likert scales for each dimension within each session, and the top three highly correlated dimensions were determined across sessions within participants. Similar to the above, each model consisted of fixed within-participant effects of thought dimension (upper vs. lower end) and the ratings of the top three correlated dimensions, as well as a random intercept of participant. If the patterns remained significant after controlling for other dimension ratings, this indicated that the observed oscillatory patterns could not be attributed to other thought dimensions. All control analyses were conducted using R (R Core Team (2022) and RStudio (2022.12.0.353)) and the following packages: Hmisc, lme4, lmertest, and tidyverse.

#### 2.7.5 Resting state

To examine the spectral signatures of each thought dimension during resting state, we first adopted an approach analogous to the analyses implemented for the self-selected task. We categorized each of the 2-second epochs across the entire 5 minutes of resting-state EEG data based on the post-rest retrospective reports, with responses to each question dichotomized into the lower end (1–3) and upper end (5–7) of the Likert scale. We then used the cluster-based permutation test to determine the oscillatory activity and its topographic distribution associated with a given thought dimension by comparing trials on the upper versus lower end of the dimension. Given there was only one experience sampling report of thoughts at the end of each resting-state session, participants may not have reported on the lower or upper end of a given thought dimension throughout any of the recording sessions. If there were no trials across sessions included on either the upper or lower end of the given thought dimension in a participant, this dimension was excluded from subsequent analyses across all participants. This resulted in the exclusion of the freely moving thought and internally oriented thought dimensions.

To directly compare the oscillatory markers of thought dimensions across conditions (task vs. rest), we compared the electrode x frequency matrices of each thought dimension during the task and resting-state components of the study. We computed a separate matrix reflecting the mean value at each electrode and frequency across recording sessions and participants for each thought dimension for the task and resting-state components. We then assessed the similarity between these matrices using the Pearson correlation coefficient.

### 2.8 Deep learning analyses

Finally, we examined whether applying the one-dimensional shallow convolution neural network (1D-SCNN), which characterizes the relatively shallow architecture in our model, on electrophysiological signals can classify each of the seven thought dimensions (Phang et al., 2020;B. Wang et al., 2023). The 1D-SCNN model is constructed by stacking multiple convolution layers, activation layers, pooling layers, and a fully connected layer to learn the interactions between the selected EEG features. This model has been successfully used for seizure detection (Salafian et al., 2023;B. Wang et al., 2023) and schizophrenia detection (Phang et al., 2020). It is also a well-validated approach for analyzing time series data and outperforms traditional machine learning algorithms in EEG classification. Specifically, the input data to our 1D-SCNN involved many features at the individual level (32 electrode x 27 frequencies = 864 features; see below). With this large number of input features, deep learning algorithms are better equipped to handle data of this size and have been shown to result in better classification performance than traditional machine learning algorithms (Mai et al., 2023).

#### 2.8.1 Extracting the features

The electrode x frequency matrix of the oscillatory component of each trial from each subject was an input feature to the 1D-SCNN. As with the above cluster-based permutation tests, we included all the channels and frequencies. For each trial, the size of the input features is number of channels (n = 32) x number of frequencies (n = 27).

##### 2.8.1.1 Building the classifier

We utilized this 1D-SCNN to learn the relationships between the extracted features from the EEG signals and thought dimensions (Phang et al., 2020;B. Wang et al., 2023). For each thought dimension, the architecture of the proposed 1D-SCNN consisted of two convolution layers, each followed by a Rectified Linear Unit (ReLU) activation function, one global average pooling layer, one dropout layer, and finally a fully connected layer followed by a classification layer with softmax function. The architecture of the 1D-SCNN model is shown inSupplementary Figure S2and its hyperparameters are reported inSupplementary Table S4. The oscillatory features were convolved in the convolution layer using multidimensional filters to learn the relationships between the extracted features from the EEG signals. This was followed by a nonlinear activation layer to produce feature maps. The inputs were then submitted to a non-learning pooling layer to perform sub-sampling on the feature maps to produce feature maps with smaller scale. This step retained the relevant information in the feature maps while ignoring information that were irrelevant for classification. After the pooling layer, the feature maps were flattened into a one-dimensional feature vector and submitted to a dropout layer to prevent overfitting. Finally, this was entered into a fully connected layer followed by a final classification layer to distinguish the two classes for each thought dimension.

The input EEG features were z-score normalized and stored in a 3D matrix representing number of trials x number of channels (n = 32) x number of frequencies (n = 27) per thought dimension. This matrix was first fed into one convolution layer in the shape of 7 kernel size * 25 filters with a stride value of 1. Next, we used one more convolution layer in the shape of 5 kernel size * 20 filters with a stride value of 1. Each convolution layer was followed by the ReLU layer for non-linear activation. Feature map outputs from the ReLU layer were then entered into a global average pooling layer. Following this was a dropout layer with a probability of 0.1, in which 10% of the outputs were removed to reduce the number of feature dimensions entering the classification layer. Finally, one fully connected layer with a size of 2 followed by a classification layer with the SoftMax function was implemented to classify the EEG signals into either class of a given thought dimension (e.g., on-task or off-task). To optimize the proposed model, we used the Adam optimizer with a learning rate of 0.0001 and the maximum epoch size is set as 100 with a batch size of 100. The binary cross-entropy loss function was used after the SoftMax layer to assess the accuracy of the 1D-SCNN model prediction by calculating how far the model’s output deviates from the ground truth as indexed by the participants’ responses to the thought probes for each dimension. The steps involved in building the classifier described above were implemented in MATLAB 2021b using the following functions: convolution1dLayer, reluLayer, globalAveragePooling1dLayer, dropoutlayer fullyConnectedLayer, softmaxLayer and classificationLayer, and trainingOptions.

##### 2.8.1.2 Evaluating the classifier

To evaluate the classifier, we implemented two approaches: a five-fold cross-validation within-participant approach and a leave-one-participant-out approach. We developed a deep learning model only for the participants who had a minimum of 50 trials in each class of a given thought dimension (e.g., at least 50 datapoints for on-task and off-task thoughts). Information regarding the number of participants included in the classification of a given thought dimension is reported inSupplementary Table S5.

For the five-fold cross-validation within-participant approach, the datapoints of each participant were randomly partitioned into k subgroups (k = 5), which is categorized into the training, testing, and validation set. In each round, one of the k subgroups served as an out-of-sample testing set while the remaining k−1 subgroups served as a training set. From the training set, 10% of the data were randomly selected for the validation set to avoid overfitting (Nahmias et al., 2020). In order to create a more balanced data set, we used the synthetic minority over-sampling technique (SMOTE;Dong et al., 2021) to oversample the minority class in the training set. Training the classification model with a balanced training set prevents the model from biasing toward the majority class and helps the model better learn the patterns representing the two classes. To assess classification performance for each thought dimension, we evaluated the model’s performance on the out-of-sample test set. As the testing set was not used for the hyperparameter tuning during training, the obtained classification performance resembles the performance based on real-world data. Specifically, we computed the mean performance by averaging across the five folds for each participant and then averaging across participants’ performance to obtain a grand average.

In addition to the within-participant approach, we also implemented the leave-one-participant-out across-participant cross-validation approach to evaluate the generalizability of the model. For this approach, each participant served as the test set in each iteration. The remaining N-1 participants served as the training set, which was split into 90% training and 10% validation to avoid overfitting. Following this, datapoints in the training set were balanced using the SMOTE technique, which were then used to train the deep learning model. Next, given that the classification performance of the leave-one-subject-out cross-validation approach is sensitive to individual level differences, we used transfer learning to adapt the deep learning model for each participant in order to improve classification performance. Transfer learning refers to transferring knowledge from a trained model to a new model. In our case, the deep learning model was first trained on all N-1 participants (as described above), after which the model was re-trained with a small amount of data (25%) from the test participant. To do so, the datapoints from the test participant was split into a 25% training and 75% testing set. Following this, the SMOTE technique was applied to the 25% training set of the test participant to balance the datapoints between classes. The balanced training set of the test participant was then used to re-train the deep learning model, which was used to evaluate the 75% testing set of the test participant. This additional step of re-training the model with a small amount of data from the test participant helps the initially trained model learn from and adapt to that participant, thereby improving the classification performance. This two-step training process was implemented N times, where N represents the number of participants. Finally, we computed the mean classification performance averaged across all iterations of N participants.

To evaluate whether the classifier accurately differentiated the two classes of each thought dimension, we used three performance metrics: Mathew’s correlation coefficient (MCC), area under the curve (AUC), and balanced accuracy (BA). The MCC metric is not susceptible to potential biases from imbalanced test data sets, whereas the AUC and BA are susceptible but were included to facilitate comparisons with past studies. For both the within-participant and across-participant approaches, we implemented two steps to test the robustness of our results. First, we implemented the aforementioned analysis for each approach 25 times and reported the mean classification performance across 25 iterations. This yields a more reliable measure of the classification performance compared with one iteration. Given the rarity of performing iterations with deep learning models with minimal existing literature to guide the optimal number of iterations, our choice of 25 iterations was primarily based on practical considerations of computation demands required to implement deep learning for all thought dimensions. Second, we performed permutation testing by randomly shuffling the labels of the thought dimensions using the same features and computing the classification performance 25 times. We first established statistical significance by comparing the mean classification performance described above against the distribution of permuted values. Given the small number of permutations however, we also implemented an alternative way to assess statistical significance to validate these results. Specifically, we implemented a Wilcoxon test (as the non-parametric version of a t-test) to compare both sets of original and permuted values from the 25 iterations.

## 3 Results

### 3.1 Thought dimension ratings and self-selected tasks

Across the seven recording sessions, participants reported a wide range of ratings for each thought dimension. The mean rating of each thought dimension across participants is depicted inFigure 1. The most commonly reported thought dimensions across sessions and participants during naturalistic tasks were freely moving, goal-oriented, and internally oriented thoughts.

**Fig. 1.**
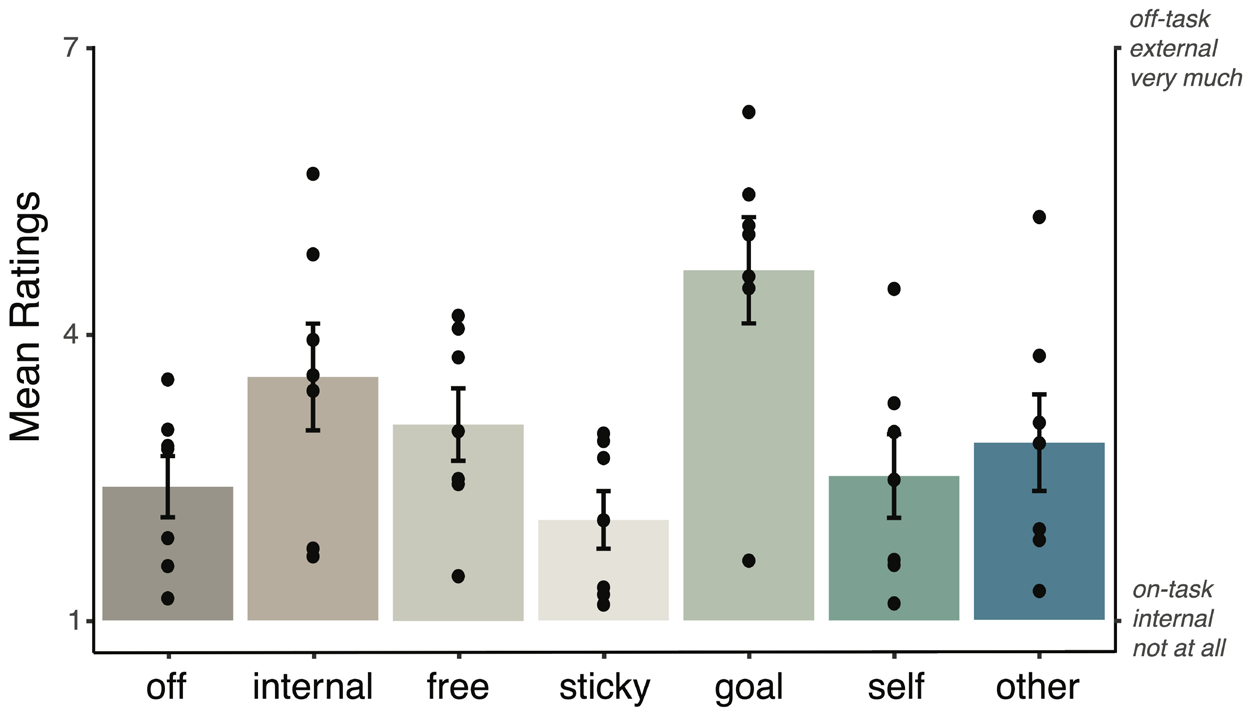
Mean rating of each thought dimension across participants.*Note*: Mean ratings ranged from 1 (not at all) to 7 (very much) for all thought dimensions, except for off-task thoughts (which ranged from very much on-task to very much off-task) and internal thoughts (which ranged from very much internal to very much external).

For the self-selected task component of the study, participants reported performing a similar set of activities. This includes reading (32%), writing or editing (14%), watching videos (19%), browsing or surfing the internet (11%), other cognitively demanding tasks (e.g., coding, playing games such as sudoku, preparing Powerpoint presentations, 23%), and others (e.g., nothing; 1%).

### 3.2 Oscillatory markers of thought dimensions during self-selected tasks

**Fig. 2.**
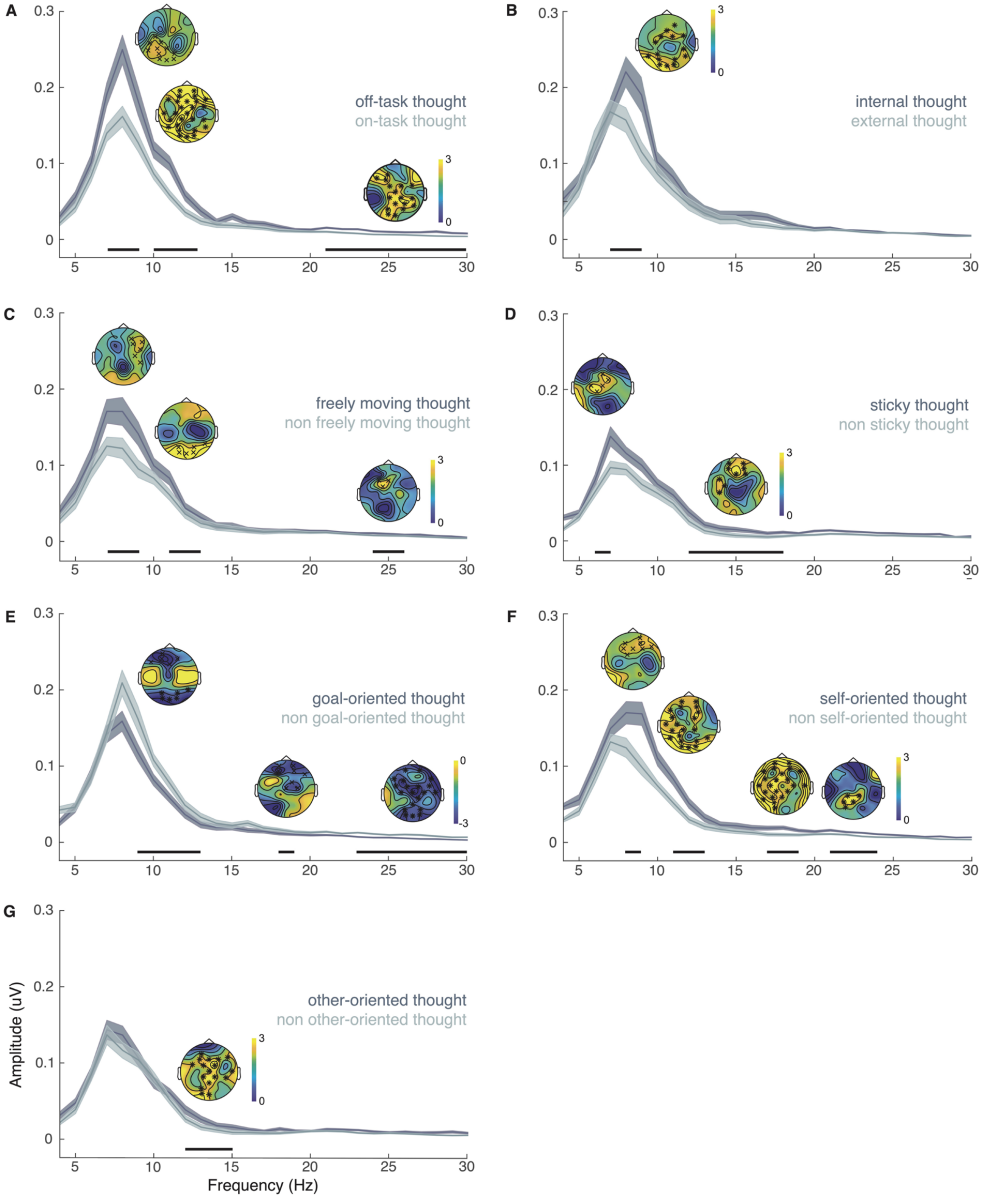
Cluster-based permutation tests on oscillatory markers of thought dimensions during self-selected tasks.*Note*: (A) Off-task thought, (B) internally oriented thought, (C) freely moving thought, (D) sticky thought, (E) goal-oriented thought, (F) self-oriented thought, and (G) other-oriented thought. Each subpanel illustrates the mean EEG spectral density of the two ends of a given thought dimension across sessions and participants (with ribbons indicating the standard errors across participants). The black horizontal lines indicate timepoints of significance between the two classes of a given thought dimension based on the cluster-based permutation tests. The x and * in the topoplots illustrate the significant topographic difference in spectral activity (with statistical t-values ranging from 0 to 3 in units of statistical scores for all thought dimensions except for goal-oriented thoughts, which ranged from -3 to 0). Warmer colors indicate greater activity at the upper end (e.g., off-task thought) relative to lower end (e.g., on-task thought) of a thought dimension, whereas cooler colors indicate greater activity during the lower end (e.g., non-goal-oriented thought) compared with upper end (e.g., goal-oriented thought) of the thought dimension.

Results from the cluster-based permutation tests assessing the oscillatory markers of each thought dimension during self-selected tasks are illustrated inFigure 2. Significant differences across electrodes and frequencies emerged for each thought dimension. Specifically, off-task thoughts were characterized by increased posterior low alpha (*p*= .020), widespread high alpha (*p*= .001), and centro-parietal beta (*p*= .001 to 043) as compared with on-task thoughts (Fig. 2A). Similarly, internally oriented thoughts showed enhanced central to posterior low alpha (*p*= .011) relative to externally oriented thoughts (Fig. 2B). We observed increased fronto-central low alpha (*p*= .009), posterior high alpha (*p*= .024), and frontal beta (*p*= .016) during freely moving thoughts compared with non-freely moving thoughts (Fig. 2C). For sticky thoughts, there was enhanced central high theta (*p*= .012) and fronto-central low beta (*p*= .001 to .038) as compared with non-sticky thoughts (Fig. 2D). Frontal and posterior alpha (*p*= .004 to .006), frontal low beta (*p*= .022), and widespread high beta (*p*= .001) were decreased when thoughts were goal oriented relative to when they were not (Fig. 2E). Self-oriented thoughts were characterized by increased frontal low alpha (*p*= .019), widespread high alpha (*p*= .001) and beta (*p*= .003 to .006) relative to thoughts not focused on the self (Fig. 2F). Finally, other-oriented thoughts showed enhanced widespread low beta (*p*= .001) compared with thoughts not focused on others (Fig. 2G).

We then assessed whether these oscillatory patterns remained significant after controlling for participant-level variance using linear mixed effects model analyses. We confirmed significance for all thought dimensions, indicating that differences in oscillatory patterns from the cluster-based permutation tests were robust against participant-level variability. Based on the same linear mixed effects models, we additionally included age and sex to examine their influence on the results. These demographic variables did not significantly predict the oscillatory patterns of any thought dimensions, and their inclusion did not change the patterns of significance of any thought dimensions. Similarly, these patterns also remained significant after controlling for the ratings of the top three thought dimensions most strongly correlated with the thought dimension of interest, suggesting that the observed patterns could not be attributed to other related thought dimensions. These control analyses are reported inSupplementary Tables S7, S8, and S9.

### 3.3 Oscillatory markers of thought dimensions during resting state

Results from the cluster-based permutation tests assessing the oscillatory markers of each thought dimension during rest are illustrated inSupplementary Figure S3. We first examined the markers of thought dimensions with sufficient data based on post-rest reports, including sticky thought, goal-oriented thought, self-oriented thought and other-oriented thought. Sticky thoughts were characterized by enhanced widespread high theta and alpha (*p*= .001) as well as reduced centro-parietal high beta (*p*= .025) compared with non-sticky thoughts (Supplementary Figure S3A). We observed decreased widespread high theta and alpha (*p*= .001) for goal-oriented thoughts compared with when thoughts were not goal oriented (Supplementary Figure S3B). For self-oriented thoughts, there was increased widespread high theta and alpha (*p*= .001) and decreased widespread low beta (*p*= .001) compared with thoughts not focused on the self (Supplementary Figure S3C). Frontocentral high theta and low alpha (*p*= .016) and widespread low beta (*p*= .003) were enhanced, whereas central high beta was reduced (*p*= .038) during other-oriented thoughts compared with when they were not (Supplementary Figure S3D).

### 3.4 Comparison of oscillatory markers across task and rest

The electrode by frequency matrices for the task and resting-state components was strongly correlated for some thought dimensions (sticky thought:*r*= 0.70,*p*< .001; self-oriented thought:*r*= 0.76,*p*< .001; other-oriented thought:*r*= 0.70,*p*< .001) and moderately correlated for others (goal-oriented thought:*r*= 0.41,*p*< .001). This indicates that although there is an overlap in oscillatory patterns of some thought dimensions across conditions, not all oscillatory patterns were comparable between naturalistic tasks and resting state.

### 3.5 Classification of thought dimensions using a deep learning algorithm

We implemented the 1D-SCNN algorithm on electrophysiological signals to evaluate the classification performance for each of the seven thought dimensions for both the five-fold cross-validation within-participant approach and the leave-one-participant-out cross-validation approach with transfer learning.Table 2reports the mean classification performance across 25 iterations for all thought dimensions for both approaches. Notably, using oscillatory features with 1D-SCNN reliably detected all seven thought dimensions at above chance levels for both within-participant (MCC = 0.22 to 0.43) and across-participant (MCC = 0.14 to 0.31) approaches. The MCC values for both approaches across all thought dimensions were statistically significant (permutation testing, all*p*’s < .05; Wilcoxon test, all*p*’s < .001). Classification performance was superior for the within-participant compared with the across-participant approach for all thought dimensions. Across both approaches, the other-oriented thought dimension had the best classification performance among all thought dimensions (within-participant: MCC = 0.43; across-participant: MCC = 0.31), followed by the goal-oriented thought dimension (within-participant: MCC = 0.35; across-participant: MCC = 0.27). Along with our oscillatory analyses reported above, these classification results indicate that the combination of 1D-SCNN and oscillatory features was capable of classifying ongoing thoughts in a naturalistic setting along seven thought dimensions. To assess the robustness of the 1D-SCNN model reported above, the training and validation classification performance for both the within- and across-participant approaches are reported inSupplementary Tables S10 and S11.

**Table 2.** Mean classification performance of thought dimensions using 1D-SCNN applied on oscillatory features for within-participant and leave-one-participant-out approaches across 25 iterations.

	Within participant			Across participant		
Thought dimension	MCC	AUC	BA	MCC	AUC	BA
on-task vs. off-task	0.25 (0.20)	0.70 (0.10)	0.61 (0.10)	0.17 (0.02)	0.63 (0.01)	0.58 (0.01)
internal vs. external	0.34 (0.27)	0.73 (0.15)	0.66 (0.14)	0.26 (0.01)	0.69 (0.01)	0.63 (0.02)
freely moving vs. not freely moving	0.22 (0.12)	0.68 (0.07)	0.60 (0.06)	0.16 (0.02)	0.63 (0.01)	0.58 (0.02)
stuck vs. not stuck	0.22 (0.13)	0.69 (0.08)	0.60 (0.14)	0.14 (0.02)	0.63 (0.01)	0.57 (0.02)
goal-oriented vs. not goal-oriented	0.35 (0.27)	0.73 (0.15)	0.66 (0.13)	0.27 (0.02)	0.69 (0.01)	0.63 (0.02)
self-oriented vs. not self-oriented	0.34 (0.27)	0.74 (0.12)	0.67 (0.07)	0.23 (0.02)	0.68 (0.01)	0.62 (0.02)
other-oriented vs. not other-oriented	0.43 (0.22)	0.80 (0.15)	0.71 (0.11)	0.31 (0.02)	0.74 (0.01)	0.66 (0.02)

*Note:*The classification performance as indexed by three performance metrics is reported for each thought dimensions. Each value represents the mean (and standard deviation) of classification performance across all 25 iterations. MCC values for all thought dimensions were statistically significant based on the Wilcoxon test and permutation testing (*p*< .05). MCC = Matthew’s Correlation Coefficient; AUC = area under curve; BA = balanced accuracy.

## 4 Discussion

In the current study, we established the electrophysiological markers of seven dimensions of ongoing thought reported during naturalistic behavior over seven recording sessions. We found that distinct oscillatory markers captured ongoing thought dimensions during both naturalistic tasks and during rest. Furthermore, deep learning models applied to oscillatory data reliably detected all seven thought dimensions at above chance levels for both within-participant and across-participant approaches. These results advance the literature in four ways. First, we established the profiles of electrophysiological signatures of multiple dimensions of ongoing thought, providing a comprehensive set of brain-to-experience mapping of various phenomenological features of thoughts. Second, capturing reliable oscillatory components allowed us to confirm that distinct patterns of neural oscillations are implicated in thought dimensions. Third, the variable patterns of electrophysiological markers of thought dimensions across task and rest suggest that while some markers are robust against the conditions in which these thoughts occur, others are not. Finally, applying convolution neural networks on electrophysiological data successfully detected the occurrence of numerous thought dimensions. We discuss the implications of these findings below.

### 4.1 Oscillatory patterns of thought dimensions

Each thought dimension was associated with a significant EEG pattern of oscillatory activity. These oscillatory patterns could not be attributed to participant-level variance and other related thought dimensions. This highlights the robustness of these oscillatory patterns, and the unique contributions offered by this study as it accounts for these potential confounding variables often overlooked in the existing literature. In addition to confirming thought dimensions consistent with past studies implemented in controlled experimental settings, we also discovered novel markers. For example, off-task thought and internally oriented thought were both associated with increased posterior low alpha indicating that posterior alpha is directly linked to processing of internal information (Kam et al., 2022;Palva & Palva, 2011). We also found that off-task thought showed widespread activity in the high alpha and beta bands often seen in resting-state patterns within the default mode network (Jann et al., 2010;G. G. Knyazev et al., 2011;Mantini et al., 2007;Neuner et al., 2014).

The three thought dimensions associated with the Dynamic Framework of Thoughts (Christoff et al., 2016) were characterized by distinct oscillatory profiles. Freely moving thought was linked to frontal low alpha activity (Kam et al., 2021). Converging evidence from creativity studies indicates that this spectral pattern is a marker of divergent thought processes (Fink et al., 2006;Lustenberger et al., 2015). We also observed posterior alpha during freely moving thought, which may reflect processing of internal information to facilitate the dynamic flow of thoughts. In contrast to thoughts that freely move from one topic to another, goal-oriented thoughts and sticky thoughts were associated with oscillatory patterns not previously reported. Specifically, goal-oriented thought was linked to reduced widespread alpha during both task and resting-state conditions and linked to reduced widespread high beta during self-selected tasks. Although frontocentral theta is often associated with cognitive control (Cavanagh & Frank, 2014), which conceptually maps onto goal-oriented thought, it is possible that goal-oriented thought engages a different process compared with traditional laboratory-based cognitive control tasks. Sticky thought was associated with increased central high theta and fronto-central low beta during self-selected tasks. Another study that examined sticky thoughts did not observe these markers during a mundane experimental task (Kam et al., 2021). One potential reason explaining this discrepancy may be that the nature of sticky thoughts (e.g., high emotional salience and relevance to priorities in life) differs between those that occurred during a controlled laboratory task versus during naturalistic tasks that may be captured by this additional marker. Importantly, this observation aligns with findings of oscillatory markers of rumination (Andersen et al., 2009;Ferdek et al., 2016), suggesting theta and beta activity may serve as a marker for highly constrained, perseverative thoughts. These observations of distinct oscillatory profiles of freely moving, goal-oriented, and sticky thoughts during naturalistic tasks provide electrophysiological evidence supporting the major tenet of the dynamic framework of thoughts proposing these as dissociable thought dimensions (Christoff et al., 2016).

Oscillatory patterns of self-oriented and others-oriented thoughts were most similar across conditions. Self-oriented thoughts were associated with increased enhanced frontal low alpha, and widespread beta during self-selected tasks. Similar and more spatially distributed patterns in high theta and alpha were observed during rest. These findings on self-oriented thought are largely consistent with the existing literature, which converge on higher frontal alpha during the processing of self-oriented information (Knyazev, 2013;Knyazev et al., 2012;Mu & Han, 2013;Travis et al., 2004). Others-oriented thoughts were associated with increased widespread low beta during tasks and rest as well as increased frontocentral high theta and low alpha during rest. Most studies that have examined others-oriented thoughts have done so in the context of theory of mind and have focused on event-related potential markers of the EEG signal that have been observed across frontal, temporal, and parietal regions (Liu et al., 2009;McCleery et al., 2011). Accordingly, although the widespread spatial distribution of the EEG signal in our data is in line with past EEG studies, our findings add to the existing literature by identifying the oscillatory correlates of others-oriented thoughts during naturalistic task settings.

Taken together, while oscillatory differences emerged across all low-frequency bands, our results point toward activity in the alpha range (8–13 Hz) playing a prominent role in numerous thought dimensions. Specifically, alpha activity was increased during off-task, internally oriented, freely moving, self-oriented and other-oriented thought, and decreased during goal-oriented thought. Each of these thought dimensions was associated with different spatial distributions in that frequency range as well as activity in other frequency bands. This suggests that alpha activity may be crucial in facilitating ongoing thoughts, in which differences in spatial patterns and co-occurrences with other frequency bands support different aspects of ongoing thoughts.

Notably, we implemented an analysis approach that captured genuine differences in oscillatory activity ruling out the possibility that the reported oscillatory patterns simply reflected the physiological component of the signal, often referred to as background noise or 1/*f*. Rather, our results suggest that oscillatory activity was present during all of these thought dimensions, highlighting their utility as electrophysiological markers in real-world and clinical applications.

### 4.2 Oscillatory markers across task and rest

The electrophysiological markers associated with some thought dimensions were comparable across self-selected tasks and resting state. Given the experimental context was different between conditions (e.g., watching a video during self-selected tasks vs. a static fixation cross at rest) and the experience sampling approach also differed between conditions (e.g., a momentary measure during tasks vs. a retrospective measure post-resting state), we anticipated that not all markers would be identical (Westlin et al., 2023). In particular, context has been implicated in objective markers of thought dimensions; for example, gaze measures of off-task thought differ as a function of the tasks performed by participants (Faber et al., 2020). The experience sampling approach may also play a role, such that the momentary measure during tasks captures the precise moments of participants’ ongoing thoughts and the corresponding oscillatory patterns during those moments, whereas the retrospective measure captures the overall level of thought dimensions across a 5-minute window and the mean oscillatory patterns averaged across the same time interval. Of importance, although the oscillatory patterns associated with a given thought dimension presumably do not change as a function of the methodological approach, the observed relationship is likely weaker in the retrospective approach relative to the experience sampling approach due to the reduced temporal precision.

Nevertheless, oscillatory markers that emerged during self-selected tasks and resting state revealed overlapping frequencies and scalp EEG regions for sticky, goal-oriented, self-oriented, and others-oriented thoughts. These oscillatory patterns in the theta and alpha range were significant in the same direction, such that a given thought dimension was similarly associated with increases or decreases in spectral activity across task and rest, whereas spectral activity in the beta range was less consistent across task and rest.

Notably, the oscillatory patterns for task and rest were strongly correlated for sticky, self-oriented, and others-oriented thoughts, indicating that these spectral signatures were less impacted by ongoing activities. In other words, whether individuals were reading, playing sudoku, or starring at a fixation cross, these oscillatory markers appeared whenever these types of thoughts occurred. The electrophysiological markers between task and rest were less robust for goal-oriented thoughts, which is a dimension that captures the constraints on thought dynamics (Christoff et al., 2016). This indicates that oscillatory markers of goal-oriented thoughts were more dependent on the activities during which these thoughts occurred. These findings collectively suggest that the oscillatory markers of some thought dimensions are generally robust against the ongoing activities and their corresponding environmental demands, whereas others (such as goal-oriented thoughts) appear to depend more on the activities during which they occur, or the experience sampling approach used to capture these thought dimensions.

### 4.3 Detection of thought dimensions using deep learning

Combining deep learning approaches with electrophysiological measures successfully predicted the occurrence of all seven dimensions of ongoing thoughts. This above-chance level performance was observed for both the five-fold within-participant cross-validation approach and the leave-one-participant-out cross-validation approach, with the within-participant approach showing superior classification performance compared with the across-participant approach for all thought dimensions. Given that individuals’ own data were used in the within-participant approach, the superior performance observed with this approach was expected. Incorporating other individuals’ data into the model in the across-participant approach inevitably introduces individual variability, thereby reducing classification performance. Notably, the deep learning model successfully predicted all thought dimensions in the across-participant approach, highlighting the generalizability of the model and its potential to be implemented in never-seen-before individuals. Collectively, these results underscore the important considerations concerning these two approaches: using individuals’ own data for classification of thought dimensions maximizes the accuracy of the prediction at the cost of having to acquire that individual’s data beforehand, whereas using a generalized model for classification may result in lower performance but does not require data obtained from any given individuals.

In terms of individual thought dimensions, classification performance for off-task thought was comparable or superior to past research for the within-participant approach but slightly inferior to past studies for the across-participant approach (Chen et al., 2022;Dong et al., 2021;Jin et al., 2019). As this was the first study to evaluate the classification performance using deep learning for all other thought dimensions, there were no previous reports with which to compare our results. Therefore, we contextualize these results by comparing them with the aforementioned studies examining the off-task thought dimension. With the exception of freely moving and sticky thought, the classification performance of all other thought dimensions for both approaches was comparable or superior to the performance of off-task thought reported in this and past studies (Chen et al., 2022;Dong et al., 2021;Jin et al., 2019;Jothiraj et al., 2025). One potential explanation for this observation is that these two thought dimensions may be associated with greater variability in electrophysiological measures within and across participants. Notably, our results suggest that applying convolution neural networks on oscillatory measures can lead to above chance detection of all seven thought dimensions. These findings along with the literature (Dhindsa et al., 2019;Dong et al., 2021;Jin et al., 2023) highlight the utility of combining machine or deep learning approaches with EEG to detect covert, mental states.

Given the paucity of studies examining oscillatory features of ongoing thoughts in a naturalistic setting, we primarily implemented data-driven approaches to facilitate a thorough examination of potential oscillatory markers of each thought dimension. Specifically, we used cluster-based permutation tests to reveal distinct oscillatory markers of thought dimensions and a deep learning algorithm without apriori channel selection using time series oscillatory data as input to detect thoughts along these dimensions. In doing so, we did not need to make any assumptions about where and how neural activity supported these thought dimensions. These two approaches also revealed complementary aspects of the results, such that the cluster-based permutation tests examined differences across participants and the deep learning analyses were implemented with both a within-participant and across-participant approach. Together, our results establish a unique set of oscillatory markers that characterize these thought dimensions, which can be used for successful detection of their occurrence during naturalistic behavior.

### 4.4 Limitations and future directions

Several points need to be taken into consideration when interpreting our results. First, our sample consisted of seven individuals, reducing the generalizability of our findings. Our participants were undergraduate and graduate students mainly recruited from the Department of Psychology and may have more background knowledge on the topic of ongoing thoughts than the general public. Although age and sex did not impact our results, our sample may be different in ways other than these demographic variables that could potentially impact the EEG results. Notably, our results are largely consistent with the literature on thought dimensions that have been previously examined involving a smaller amount of individual-level data from a larger participant pool, suggesting that our markers generalize across individuals beyond our sample. We also found that the oscillatory markers remained significant in differentiating the presence of thought dimensions after statistically controlling for participant-level variance, suggesting the observed differences could not be attributed to variance across participants. Instead of acquiring a single data set from more participants, this study design prioritized acquiring sufficient data from each participant (i.e., 7 recording sessions from 7 participants resulting in 49 data sets) to obtain reliable estimates of each electrophysiological marker for each of the 7 thought dimensions within participants. This approach enabled us to optimally address our research aims concerning oscillatory markers of multiple thought dimensions.

Another point of consideration concerns potential overlap in thought dimensions. With our current approach of examining each thought dimension separately, it is possible that electrophysiological markers of one thought dimension may reflect markers of another dimension if those two ratings are highly correlated (for example, if off-task thought was always rated as internally oriented, then the markers for both thought dimensions would be identical). In statistically controlling for ratings of the three highest correlated dimensions, we found that the oscillatory markers of a given thought dimension remained significant. This suggests that although some thought dimensions were correlated with each other, the ratings of the other dimensions did not significantly contribute to the oscillatory markers of the target thought dimension. This is further corroborated by the distinct oscillatory patterns observed for each thought dimension. A novel way to address the conceptual overlap in thought dimensions is to directly model these co-occurrences. Given that recent studies have implemented dimension reduction techniques on multi-dimensional experience sampling data to highlight thought patterns rather than focusing on single dimensions separately (Konu et al., 2020;Mckeown et al., 2021;Turnbull et al., 2021), future studies may implement these techniques to explore electrophysiological signatures of thought patterns.

Finally, although our convolution neural network successfully detected all seven thought dimensions above chance, classification performance can be enhanced through other means. For example, future work can improve classification performance of these thought dimensions by combining the 1D-SCNN with other deep learning architectures. Specifically, although we implemented a deep learning algorithm, it was a shallow network. This suggests that our approach may not have captured more complex patterns in the data that are facilitated by deeper neural networks. Another approach to enhancing classification performance involves expanding on the number of channels (e.g., 64 or 128 channels) if computational demand is not an important consideration. A related approach involves implementing deep learning on the raw EEG signal, as it may carry unique information not captured by a single frequency band and it is less computationally demanding than feature extraction which is an important consideration for real-time implementation (Lawhern et al., 2018). This combination of EEG and deep learning approach has important clinical implications as they can be used for detecting relevant thought dimensions in different groups, such as freely moving thought in attention-deficit hyperactivity disorder or sticky thought in depression (Kucyi et al., 2023). In addition, enhanced classification performance could suggest that future studies can rely solely on electrophysiological data to detect a given thought dimension without the use of thought probes, circumventing the concern that the appearance of thought probes may interrupt ongoing thought.

## 5 Conclusion

In summary, our study established the oscillatory markers of a comprehensive set of dimensions of ongoing thoughts during naturalistic behavior and demonstrated that oscillatory features can be extracted in deep learning architectures to detect thoughts across various dimensions. The implications are twofold. First, our findings have imminent real-world applications, as they represent a crucial first step toward predicting the occurrence of a given thought dimension in everyday life and in real time. One potential major implication of establishing the optimal EEG features associated with thought dimensions is the opportunity to regulate them. For instance, by detecting these thought dimensions in real time, future research can then explore the potential to increase adaptive thought (e.g., goal-oriented or freely moving thoughts) and decrease maladaptive thought (e.g., sticky or ruminative thoughts) dimensions using techniques such as behavioral intervention, neurofeedback, or neurostimulation. Second, these findings have important clinical implications for establishing atypical biomarkers of thought dimensions in different populations, which may be useful for differentiating individuals based on a simple experimental design with minimal task constraints. Both real-world and clinical applications highlight meaningful avenues for future research concerning the electrophysiological signatures of ongoing thoughts.

## Supplementary Material

Supplementary Material

## Data Availability

The data and code that support the findings of this study are available onhttps://osf.io/9hrny/.
