# Supplementary material for: Electrophysiological signatures of ongoing thoughts during naturalistic behavior
Source: Imaging Neurosci (Camb). 2025 Jun 5;3:IMAG.a.20. doi: 10.1162/IMAG.a.20 (PMC12319872; doi:10.1162/IMAG.a.20)
Supplement: Supplementary Material [file imag.a.20_supp.pdf]

Electrophysiological Signatures of Ongoing Thoughts during Naturalistic Behavior

Supplementary Materials

Julia W. Y. Kam, Tarannum Rahnuma, Sairamya Nanjappan Jothiraj,

Alexandra A. Ouellette-Zuk, Robert T. Knight

Supplementary Methods

Statistical Analyses

To examine the spectral signatures of each thought dimension during self-selected tasks, we first categorized EEG data based on participants' responses to the thought probes. Specifically, their responses to each question were dichotomized into two groups separating responses on the lower end (1-3) and upper end (5-7) of the Likert scale and discarding the middle response (4). Our choice to dichotomize the response is based on a pragmatic consideration concerning the cluster-based permutation tests (CBPT) within the Fieldtrip toolbox. This CBPT approach is advantageous because it allows for the examination of conditional differences while correcting for multiple comparisons across the multi-dimensional nature of EEG data (e.g., electrodes, time, frequency, trials, participants). Unfortunately, although the CBPT function allows one to examine the relationship between EEG data and a continuous behavioral variable, it is limited to analysis at the data set level. For example, we can assess the relationship between 49 EEG data sets and 49 thought dimension ratings linked to each data set. This means the input for the ratings would have to be an aggregate value across all thought probes within a session (representing a data set), thereby removing the primary benefits of an experience sampling approach that captures fluctuations of ongoing thoughts in the moment at each thought probe throughout the course of a session. Not only does this remove important information about ongoing thoughts and their corresponding EEG patterns at precise moments in time, but it also correspondingly limits the interpretation to the following: EEG sessions where thoughts were rated on average as more task-unrelated showed higher levels of widespread alpha in general averaged across the session. This type of interpretation is less precise and does not allow capture of the temporally precise association between EEG markers and ongoing thoughts that our current approach enables. Specifically, our current approach facilitates this type of interpretation: there is widespread alpha *during* periods of task-unrelated thought. In summary, although we potentially sacrificed some information in the continuous ratings by dichotomizing the responses on a multi-point Likert scale, we were able to implement the cluster-based permutation test to determine the oscillatory markers of thoughts dimensions and we also gained the ease of interpretation and understanding of our results through this approach. Therefore, instead of examining the full range of response on the 7-point Likert scale, we dichotomized the responses instead.

In addition to examining oscillatory patterns of thought dimensions at the group level, we attempted to address this question at the individual level. Although we have acquired seven data sets per participant, that does not yield sufficient power to detect statistical significance. A power analysis with a two-tailed alpha of 0.05, power of 0.90 and an estimated effect size of 0.62 (e.g., Arnau et al., 2020; Compton et al., 2019; Dhindsa et al., 2019) suggests a minimum of 30 data sets would be necessary. Nevertheless, we implemented post hoc analyses involving the same linear mixed effects model reported in the manuscript for each participant to determine whether these oscillatory patterns may be detected at the single subject level. Each model consisted of a fixed within-participant effect of thought dimension (upper versus lower end) and a random effect of recording session (1 to 7). The dependent variable was the overall mean of the EEG datapoints within the significant electrode x frequency clusters that emerged from the cluster-based permutation test averaged within sessions for each participant. Not all participants had sufficient data for a given dimension for the analysis to be implemented; therefore, some participants were excluded from the analysis of a given thought dimension. With only up to seven sessions of data for each participant in each model, these analyses did not have sufficient power to detect significant effects (as reported above). Therefore, interpretation of these results should take this issue into consideration.

Supplementary Results

Oscillatory Markers of Thought Dimensions using 8 second Time Window During Tasks

In addition to categorizing EEG data within the 12 second time window preceding a thought probe, we also tested whether a shorter (i.e., 8 seconds) and longer (i.e., 16 seconds) time window led to the same results. For the 8 second time window, significant differences across electrodes and frequencies emerged for each thought dimension in a similar pattern and direction as the analysis with the 12 second time window, with the main exception of internally oriented thoughts. Specifically, off-task thoughts were characterized by increased central and posterior low alpha (p = .010), fronto-centro-parietal high alpha (p = .004 to .032), and centro-parietal beta (p = .002 to 004) as compared to on-task thoughts. Unlike the 12 second time window results, internally and externally oriented thoughts did not show significant differences. We observed increased posterior high alpha (p = .024) during freely moving thoughts compared to non-freely moving thoughts. Although increased fronto-central alpha was observed during freely moving thought as with the 12 second time window analysis, this difference only trended (p = .079 to .081). For sticky thoughts, there was enhanced central high theta (p = .015), widespread low beta (p = .001 to .035) and central high beta (p = .024) as compared to non-sticky thoughts. Fronto-centro-parietal high alpha (p = .003), frontal low beta (p = .045) and centro-parietal high beta (p = .001 to .002) were decreased when thoughts were goal-oriented relative to when they were not. Self-oriented thoughts were characterized by increased frontal and parietal low alpha (p = .020 to .048), fronto-centro-parietal high alpha (p = .001) and low beta (p = .001) as well as centro-parietal high beta (p = .005 to .011) relative to thoughts not focused on the self. Finally, other-oriented thoughts showed enhanced posterior low beta (p = .041) compared to thoughts not focused on others.

Oscillatory Markers of Thought Dimensions using 16 second Time Window During Tasks

For the 16 second time window, significant differences across electrodes and frequencies emerged for each thought dimension in a similar pattern and direction as the analysis with the 12 second time window, with the main exception of others-oriented thoughts. Specifically, off-task thoughts were characterized by increased widespread alpha (p = .001 to .019), and fronto-centro-parietal beta (p = .001 to 040) as compared to on-task thoughts. Similarly, internally oriented thoughts showed enhanced central to parietal low alpha (p = .038) relative to externally oriented thoughts. We observed increased widespread alpha (p = .002) during freely moving thoughts compared to non-freely moving thoughts, but not frontal beta which was significant in the 12 second time window analysis. For sticky thoughts, there was

enhanced fronto-central high theta (*p* = .004), central low beta (*p* = .004) and high beta (p = .030) as compared to non-sticky thoughts. Frontal and posterior alpha (*p* = .025), fronto-central low beta (*p* = .009) and widespread high beta (*p* = .001) were decreased when thoughts were goal-oriented relative to when they were not. Self-oriented thoughts were characterized by increased fronto-centro-parietal high alpha (*p* = .001), fronto-centro-parietal low beta (*p* = .002) and centro-parietal high beta (*p* = .006 to .023) relative to thoughts not focused on the self. Finally, although other-oriented thoughts showed increased central low beta compared to thoughts not focused on others, this difference only trended (*p* = .084).

Oscillatory Markers of Visual and Auditory Thought Dimensions During Tasks

Results from the cluster-based permutation tests assessing the oscillatory markers of the visual and auditory modalities during self-selected tasks are illustrated in Supplementary Figure 1. Similar to the other dimensions, participants rated the extent to which their thoughts were in the visual and auditory modality on a 7-point Likert scale ranging from 1=not at all to 7=very much. The mean ratings for visual and auditory thoughts were 3.31 (SEM = 0.01) and 3.57 (SEM = 0.01) respectively. For visual thoughts, there were no significant clusters. For auditory thoughts, there was increased posterior alpha (p = .043) as compared to non-auditory thoughts.

Individual Level Oscillatory Markers of Thought Dimensions

Supplementary Table S6 reports the individual level linear mixed effects analyses examining the oscillatory measures of each thought dimension. For the off-task, sticky, and goal-oriented thought dimensions, all but one participant showed a significant effect of dimension. For the freely moving dimension, four participants showed a significant effect of dimension. For the remaining thought dimensions (internally-oriented, self-oriented, and others-oriented), a significant effect of dimension was reported in at least two participants. Importantly, for all the significant effects, the effect observed at the individual level was in the same direction as the effect observed at the group level. In four instances, the individual level pattern showed the opposite direction as the group level pattern (based on the beta value). Although the effect of dimension was not significant in these models, it highlights individual differences in the oscillatory markers of thought dimensions. We caution any interpretation of these results given these analyses were underpowered.

In addition to these analyses, we also presented the mean spectral power within significant clusters based on cluster-based permutation tests, extracted for each level of the dimension averaged across sessions within participants. Supplementary Figure S3 illustrates

the oscillatory power of both levels of each dimension for each participant, demonstrating that most participants showed the same oscillatory patterns for each of the seven dimensions.

Supplementary Table S1

Definitions and Example Scenarios of All Multi-Dimensional Experience Sampling Questions.

What is your current task?	Definition:<br>"Task" refers to whatever it is you're supposed to be doing. This could be actual work or doing a google search or watching a video. This doesn't need to be a physical or "productive" task (e.g., if you're intending to daydream, then your task is daydreaming).
	Response:<br>Open-ended response.
To what extent were your thoughts on-task versus off-task?	Definition:<br>Based on the task you listed in the previous question, were you paying attention to the task or not?
	Examples:<br>1 = very much on-task, if you were completely focused on finishing your assigned task for class (your task).<br>7 = very much off-task, if your thoughts were not focused on reading your e-book (your task) at all, and instead focused on planning your next vacation
To what extent were your thoughts focused on your inner thoughts or external stimuli?	Definition:<br>Internal implies that your thoughts were internally focused, for example if you were doing a math problem in your head or recalling memories of past vacations. External implies that your thoughts were focused on an external stimulus, either visual, auditory or tactile like the image you're seeing on the screen, or the sensation of the EEG cap on your head.
	Examples:<br>1 = very much internal, if your thoughts are exclusively focused internally, to the exclusion of any external stimuli<br>7 = very much external, if your thoughts were exclusively focused on an external stimulus over any internal thoughts
To what extent were your thoughts freely moving?	Definition:<br>Freely moving means your thoughts wandered through different topics with no overarching purpose or direction, and thoughts were not focused on anything for too long.
	Examples:<br>1 = not at all, if your thoughts were strictly focused on one topic (e.g., the pandemic or career plans).<br>7 = very much, if your thoughts wandered swiftly from your future plans to the concept of equality to childhood memories to your friend's wedding and did so for the entire 5 minutes.

To what extent were your thoughts goal-directed on a topic?	Definition:<br>Goal-directed means that your thoughts are focused on an overarching goal, and that when you have thoughts that distract you from your goal, you bring your mind back on track<br>Examples:<br>1 = not at all, if you were watching a video or browsing the internet with no particular goal, and your attention to these activities wanders aimlessly<br>7 = very much, if you were very focused on imagining the sequence of movements in your dance routine as rehearsal for your upcoming dance recital
To what extent did you have difficulty disengaging from your thoughts?	Definition:<br>An inability to disengage means that you can't help but think about that topic, and can't stop if you want to; your thoughts are drawn to this topic, whether or not you're actively trying to think about it.<br>Examples:<br>1 = not at all, if you were not stuck on any topic and were able to easily stop thinking about it and move on to another thought.<br>7 = very much, if you couldn't help but think about your sibling who got into a car accident. No matter how hard you tried your thoughts were stuck on this topic for the entire 5 minutes.
To what extent were your thoughts about yourself?	Definition:<br>Thoughts are about yourself.<br>Examples<br>1 = not at all, if you were only thinking about your sibling and their dog and not thinking about yourself at all.<br>7 = very much, if your thoughts were focused on yourself, like what you want to do in a future career, and not related to someone else.
To what extent were your thoughts about another person/other people?	Definition:<br>Thoughts are about others.<br>Examples:<br>1 = not at all, if your thoughts were focused on yourself, or an abstract topic like art or university policy, and not related to someone else.<br>7 = very much, if you were thinking about someone else, like your friend or parents, and not yourself.

Note. These definitions and examples were provided to participants to ensure they understood the meaning of each question asked at thought probes. The questions here were asked during the task and are identical in content as those asked during resting state, with two exceptions. First, the first two questions were only asked during the task condition and not the resting state condition. Second, instead of asking “to what extent” participants experienced a thought dimension, they were asked “how often” they experienced a thought dimension during the

resting state, which they responded to on a 7-point Likert scale, ranging from not at all to all the time. Given that a single thought probe was presented at the end of the resting state session, the slight modification in the response options were meant to capture the frequency of these thought dimensions across the entire resting state session (as opposed to their momentary thoughts at the end of the session).

Supplementary Table S2

Breakdown of the number of artifact-free trials in total and included in subsequent analysis.

A. Total number of trials recorded for each participant at each session								
Participant	EEG Session							
	1	2	3	4	5	6	7	Total
1	2621	2530	2342	2404	2370	1903	2142	16312
2	2531	2478	2258	2422	2291	2258	2290	16528
3	2414	2286	2492	2345	2235	2180	2229	16181
4	2598	2832	2511	2219	2418	2470	2426	17474
5	2977	2522	2573	2627	2549	2388	2475	18111
6	2657	2454	2443	2428	2305	2333	2291	16911
7	2573	2566	2456	2430	2224	2512	2447	17208

B. Number of trials included in subsequent analysis for each participant and session								
Participant	EEG Session							
	1	2	3	4	5	6	7	Total
1	181	194	160	178	182	147	169	1211
2	187	202	184	198	193	206	205	1375
3	186	172	182	184	158	177	186	1245
4	182	204	165	180	209	196	199	1335
5	178	139	154	128	168	141	168	1076
6	188	204	207	185	199	174	188	1345
7	153	176	171	130	144	179	183	1136

Note. The total number of trials for each participant and session refers to trials spanning each experimental session (as reported in A). Based on the experience sampling approach, only the 6 trials falling within 12 seconds preceding the experience sampling probe were included in subsequent analysis (as reported in B).

Supplementary Table S3

Breakdown of the number of artifact-free trials for the upper and lower end of thought dimensions for each participant.

Thought Dimension	1	2	3	4	5	6	7
Task-relatedness							
off-task	335	0	225	80	332	48	287
on-task	876	1375	1020	1255	744	1297	849
Internal-External							
Internal	759	555	1064	36	243	36	353
external	405	685	183	1284	821	1276	656
Freely moving							
free moving	655	45	314	77	519	12	311
not freely moving	580	1246	632	1183	465	1360	869
Sticky							
Sticky	298	0	277	62	282	18	149
not sticky	873	1379	901	1333	926	1360	1076
Goal-oriented							
goal-oriented	810	121	1014	1174	1023	1031	721
not goal-oriented	427	1231	200	173	108	47	409
Self-oriented							
self-oriented	411	6	725	72	297	18	52
not self-oriented	855	1373	401	1251	867	1362	1200
Other-oriented							
other-oriented	393	101	410	998	442	134	36
not other-oriented	859	1221	856	258	747	1220	1219

Note. The total number of trials across sessions for each participant (reported in each column) for the upper and lower ends of each thought dimension.

Supplementary Table S4

Configuration of the 1 Dimensional – Convolution Neural Network

Layer	Type	Number	Size	Stride	Activation	Dropout	Parameters
1	Input	-	32*27	-	-	-	
2	Convolution layer	25	7*1	1	ReLU	-	5625
3	Convolution layer	20	5*1	1	ReLU	-	5625
4	Global average pooling layer	-	-	-	-	0.1	0
5	Fully connected layer	2	-	-	ReLU	-	42
6	Output	2	-	-	Softmax	-	0

Note. ReLU = the Rectified Linear Unit

Supplementary Table S5

Participants Included in Classification of Thoughts Dimensions

Partici pant	off-task thought	internally oriented thought	freely moving thought	sticky thought	goal-oriented thought	self-oriented thought	other-oriented thought
1	✓	✓	✓	✓	✓	✓	✓
2	✓	✓	✓	✓		✓	
3	✓	✓	✓	✓	✓	✓	✓
4	✓	✓	✓	✓	✓	✓	✓
5	✓	✓	✓	✓		✓	✓
6				✓	✓		
7	✓	✓	✓		✓	✓	✓

Supplementary Table S6

Individual-level Analyses of Thought Dimension predicting Oscillatory Measures using Linear Mixed Effects Model Analyses.

Thought Dimension	Participant	β	SE	95% CI	$\chi^2(1)$	*p*
off-task thought						
	S1	0.03	0.013	[0.003, 0.05]	5.30	.021
	S3	0.02	0.009	[0.003, 0.04]	6.13	0.01
	S4	0.01	0.002	[0.01, 0.02]	32.82	<.001
	S5	0.01	0.010	[-0.01, 0.03]	0.63	.423
	S6	0.04	0.009	[0.02, 0.07]	23.37	<.001
	S7	0.01	0.003	[0.003, 0.01]	9.40	.002
internally oriented thought						
	S1	0.01	0.008	[-0.01, 0.02]	0.56	.454
	S2	-0.02	0.011	[-0.04, 0.01]	2.21	.137
	S3	0.16	0.059	[0.04, 0.27]	6.85	.009
	S4	0.02	0.002	[0.02, 0.02]	118.9	<.001
	S5	-0.01	0.017	[-0.04, 0.02]	0.26	.612
	S6	0.43	0.09	[0.22, 0.63]	21.14	<.001
	S7	0.01	0.006	[-0.003, 0.02]	2.29	.131
freely moving thought						
	S1	0.02	0.007	[0.002, 0.03]	5.84	.016
	S2	0.02	0.007	[0.00, 0.03]	4.94	0.03
	S3	0.10	0.020	[0.06, 0.14]	25.65	<.001
	S4	0.02	0.007	[0.005, 0.03]	6.59	.010
	S5	0.002	0.009	[-0.02, 0.02]	0.04	.845
	S7	0.01	0.007	[0.002, 0.03]	3.10	.078
sticky thought						
	S1	0.01	0.003	[0.00, 0.01]	4.89	.027
	S3	0.04	0.014	[0.01, 0.06]	7.05	.008
	S4	0.03	0.005	[0.01, 0.04]	27.40	<.001
	S5	0.01	0.005	[-0.002, 0.02]	2.93	.090
	S7	0.01	0.004	[0.00, 0.02]	4.57	.032
goal-oriented thought						
	S1	-0.02	0.007	[-0.03, -0.003]	5.92	.015
	S2	0.003	0.007	[-0.01, 0.02]	0.16	.690
	S3	-0.01	0.007	[0.03, -0.00]	3.79	.052
	S4	-0.01	0.004	[-0.02, -0.004]	8.56	.003
	S5	-0.01	0.004	[-0.02, -0.002]	6.08	.014
	S6	-0.01	0.004	[-0.02, -0.003]	9.05	.003
	S7	-0.01	0.003	[-0.01, 0.00]	4.66	.031
self-oriented thought						
	S1	0.02	0.001	[-0.002, 0.04]	3.42	.064
	S3	0.02	0.010	[-0.002, 0.04]	3.55	.060

	S4	0.02	0.002	[0.01, 0.02]	43.21	<.001
	S5	0.01	0.010	[-0.01, 0.03]	1.82	.177
	S6	0.04	0.022	[-0.01, 0.09]	3.32	.065
	S7	0.02	0.003	[0.01, 0.02]	32.19	<.001
other-oriented thought						
	S1	0.01	0.005	[-0.004, 0.02]	1.80	.180
	S2	0.01	0.004	[-0.002, 0.01]	2.06	.151
	S3	0.01	0.003	[0.003, 0.02]	10.15	.001
	S4	-0.001	0.003	[-0.01, 0.06]	0.04	.842
	S5	0.004	0.004	[-0.005, 0.01]	0.96	.327
	S7	0.03	0.007	[0.02, 0.05]	23.43	<.001

Note. β=beta coefficient. SE=standard error of the mean and 95% CI = confidence interval, associated with the standardized coefficient. p-value associated with the χ^2-statistic, which tests the current model against a null model without the independent variable of interest. Each set of rows represent separate analyses for each participant for a given thought dimension, which includes the thought dimension condition (e.g., off-task versus on-task) as fixed effects and a random effect of session as independent variables. Given the small number of data sets within a given participant for each analysis, we caution that meaningful interpretation of the results is not possible at the single individual level (as each model lacked sufficient power to reliably detect effects of interest).

Supplementary Table S7

Control Analyses of Thought Dimension predicting Oscillatory Measures while accounting for Age and Sex using Linear Mixed Effects Model analyses.

IV		β	SE	95% CI	$\chi^2(1)$	*p*
off-task thought						
	condition	0.02	0.004	[0.01, 0.03]	24.15	<.001
	age	0.001	0.002	[-0.002, 0.005]	0.57	.450
	sex	0.005	0.015	[-0.02, 0.03]	0.11	.739
internally oriented thought						
	condition	0.07	0.026	[0.02, 0.12]	6.78	.009
	age	0.002	0.007	[-0.01, 0.02]	0.09	.759
	sex	0.04	0.062	[-0.08, 0.16]	0.34	.561
freely moving thought						
	condition	0.03	0.008	[0.01, 0.04]	15.19	<.001
	age	-0.001	0.002	[-0.01, 0.003]	0.21	.647
	sex	-0.02	0.021	[-0.06, 0.02]	0.77	.381
sticky thought						
	condition	0.02	0.004	[0.01, 0.02]	22.52	<.001
	age	0.001	0.001	[-0.001, 0.004]	1.03	.310
	sex	0.002	0.010	[-0.02, 0.02]	0.03	.857
goal-oriented thought						
	condition	-0.01	0.002	[-0.01, -0.01]	26.80	<.001
	age	0.00	0.001	[-0.001, 0.001]	0.03	.856
	sex	0.00	0.005	[-0.01, 0.01]	0.00	.999
self-oriented thought						
	condition	0.02	0.003	[0.01, 0.03]	28.66	<.001
	age	-0.001	0.001	[-0.002, 0.001]	0.25	.617
	sex	-0.02	0.010	[-0.03, 0.004]	2.79	.095
other-oriented thought						
	condition	0.01	0.003	[0.005, 0.02]	14.99	<.001
	age	0.00	0.001	[-0.001, 0.002]	0.04	.848
	sex	-0.002	0.007	[-0.02, 0.01]	0.12	.728

Note. IV=independent variables. β=beta coefficient. SE=standard error of the mean and 95% CI = confidence interval, associated with the standardized coefficient. p-value associated with the χ^2-statistic, which tests the current model against a null model without the independent variable of interest. Each set of rows represent separate analyses for each thought dimension, which includes the thought dimension condition (e.g., off-task versus on-task), age, and sex as fixed effects and a random effect of session nested within participant as IVs. Given the small sample size, it is not particularly meaningful to examine age and sex effects; nonetheless, these results indicate that age and sex did not predict oscillatory patterns, nor did they impact the pattern of significance of results.

Supplementary Table S8

Control Analyses of Thought Dimension predicting Task-based Oscillatory Measures while accounting for Participant Level Variance using Linear Mixed Effects Model analyses.

IV	β	SE	95% CI	$\chi^2(1)$	*p*
off-task thought	0.02	0.004	[0.01, 0.03]	24.44	<.001
internally oriented thought	0.07	0.025	[0.02, 0.12]	7.22	.007
freely moving thought	0.03	0.008	[0.01, 0.04]	15.95	<.001
sticky thought	0.02	0.003	[0.01, 0.02]	22.52	<.001
goal-oriented thought	-0.01	0.002	[-0.02, -0.01]	27.92	<.001
self-oriented thought	0.02	0.004	[0.01, 0.03]	27.41	<.001
other-oriented thought	0.01	0.003	[0.01, 0.02]	15.75	<.001

Note. IV=independent variables. β=beta coefficient. SE=standard error of the mean and 95% CI = confidence interval, associated with the standardized coefficient. p-value associated with the χ^2-statistic, which tests the current model against a null model without the independent variable of interest. Each row represents separate analyses for each thought dimension, which includes the thought dimension condition (e.g., off-task versus on-task) as a fixed effect and a random effect of session nested within participant as IVs.

Supplementary Table S9

Control Analyses of Thought Dimension predicting Task-based Oscillatory Measures while accounting for Ratings of Highly Correlated Dimensions using Linear Mixed Effects Model analyses.

IV	β	SE	95% CI	$\chi^2(1)$	*p*
off-task thought					
condition	0.02	0.004	[0.01, 0.03]	24.44	<.001
control dimension 1	0.001	0.006	[-0.01, 0.01]	0.05	.826
control dimension 2	0.004	0.006	[-0.01, 0.01]	0.65	.422
control dimension 3	-0.003	0.005	[-0.01, 0.01]	0.37	.546
internally oriented thought					
condition	0.07	0.025	[0.02, 0.12]	7.22	.007
control dimension 1	0.01	0.033	[-0.06, 0.07]	0.03	.861
control dimension 2	-0.01	0.022	[-0.03, 0.06]	0.29	.589
control dimension 3	-0.004	0.020	[-0.04, 0.04]	0.04	.849
freely moving thought					
condition	0.03	0.007	[0.01, 0.04]	15.95	<.001
control dimension 1	-0.01	0.008	[-0.02, 0.01]	0.52	.471
control dimension 2	0.001	0.007	[-0.01, 0.01]	0.03	.873
control dimension 3	0.001	0.006	[-0.01, 0.01]	0.06	.809
sticky thought					
condition	0.02	0.003	[0.01, 0.02]	22.52	<.001
control dimension 1	0.003	0.007	[-0.01, 0.02]	0.15	.701
control dimension 2	0.004	0.008	[-0.01, 0.02]	0.27	.601
control dimension 3	0.004	0.005	[-0.01, 0.02]	0.54	.461
goal-oriented thought					
condition	-0.01	0.002	[-0.02, -0.01]	27.92	<.001
control dimension 1	-0.002	0.003	[-0.01, 0.003]	0.71	.401
control dimension 2	0.003	0.003	[-0.002, 0.01]	1.26	.262
control dimension 3	0.001	0.002	[-0.003, 0.01]	0.37	.545
self-oriented thought					
condition	0.02	0.004	[0.01, 0.03]	27.41	<.001
control dimension 1	-0.01	0.005	[-0.01, 0.01]	0.83	.364
control dimension 2	-0.003	0.004	[-0.01, 0.005]	0.44	.508
control dimension 3	0.001	0.003	[-0.01, 0.01]	0.67	.796
other-oriented thought					
condition	0.01	0.002	[0.01, 0.02]	15.75	<.001
control dimension 1	0.002	0.004	[-0.005, 0.01]	0.38	.536
control dimension 2	-0.01	0.002	[-0.01, -0.001]	6.15	.013
control dimension 3	0.001	0.003	[-0.004, 0.01]	0.08	.774

Note. IV=independent variables. β=beta coefficient. SE=standard error of the mean and 95% CI

= confidence interval, associated with the standardized coefficient. p-value associated with the χ^2-statistic, which tests the current model against a null model without the independent variable of interest. Each set of rows represents separate analyses for each thought dimension, which includes the thought dimension condition (e.g., off-task versus on-task; to parallel the cluster-based permutation test approach) and the ratings of three highly correlated dimensions (i.e., on a 7-point Likert scale) as a fixed effects and a random effect of session nested within participants as IVs.

Supplementary Table S10

Mean Classification Performance across 25 Iterations for Training and Validation Sets for the Five-Fold Cross-Validation Within Participant Approach

	Training Set			Validation Set		
**Thought Dimension**	**MCC**	**AUC**	**BA**	**MCC**	**AUC**	**BA**
on-task vs off-task	0.79 (0.11)	0.94 (0.04)	0.88 (0.06)	0.27 (0.19)	0.71 (0.09)	0.62 (0.10)
internal vs external	0.70 (0.21)	0.90 (0.10)	0.84 (0.10)	0.36 (0.27)	0.75 (0.14)	0.67 (0.14)
freely moving vs not freely moving	0.64 (0.18)	0.87 (0.07)	0.81 (0.08)	0.24 (0.11)	0.70 (0.07)	0.61 (0.06)
stuck vs not stuck	0.81 (0.12)	0.94 (0.04)	0.89 (0.07)	0.25 (0.12)	0.70 (0.08)	0.61 (0.07)
goal-oriented vs not goal-oriented	0.79 (0.20)	0.93 (0.08)	0.89 (0.10)	0.37 (0.27)	0.75 (0.14)	0.67 (0.14)
self-oriented vs not self-oriented	0.85 (0.12)	0.97 (0.04)	0.93 (0.06)	0.34 (0.27)	0.76 (0.11)	0.67 (0.14)
other-oriented vs not other-oriented	0.78 (0.19)	0.94 (0.08)	0.89 (0.10)	0.45 (0.22)	0.82 (0.14)	0.72 (0.11)

Note. The classification performance as indexed by three performance metrics are reported for each thought dimension for the training set (left) and validation set (right). Each value represents the mean (and standard deviation) of classification performance across all 25 iterations. MCC = Matthew's Correlation Coefficient; AUC = Area Under Curve; BA = Balanced Accuracy.

Supplementary Table S11

Mean Classification Performance across 25 Iterations for Training and Validation Sets for the Leave-One-Participant-Out Cross-Validation Approach

	Training Set			Validation Set		
**Thought Dimension**	**MCC**	**AUC**	**BA**	**MCC**	**AUC**	**BA**
on-task vs off-task	0.79 (0.11)	0.94 (0.04)	0.88 (0.06)	0.27 (0.19)	0.71 (0.09)	0.62 (0.10)
internal vs external	0.70 (0.21)	0.90 (0.10)	0.84 (0.10)	0.36 (0.27)	0.75 (0.14)	0.67 (0.14)
freely moving vs not freely moving	0.64 (0.18)	0.87 (0.07)	0.81 (0.08)	0.24 (0.11)	0.70 (0.07)	0.61 (0.06)
stuck vs not stuck	0.81 (0.12)	0.94 (0.04)	0.89 (0.07)	0.25 (0.12)	0.70 (0.08)	0.61 (0.07)
goal-oriented vs not goal-oriented	0.79 (0.20)	0.93 (0.08)	0.89 (0.10)	0.37 (0.27)	0.75 (0.14)	0.67 (0.14)
self-oriented vs not self-oriented	0.85 (0.12)	0.97 (0.04)	0.93 (0.06)	0.34 (0.27)	0.76 (0.11)	0.67 (0.14)
other-oriented vs not other-oriented	0.78 (0.19)	0.94 (0.08)	0.89 (0.10)	0.45 (0.22)	0.82 (0.14)	0.72 (0.11)

Note The classification performance as indexed by three performance metrics are reported for each thought dimension for the training set (left) and validation set (right). Each value represents the mean (and standard deviation) of classification performance across all 25 iterations. MCC = Matthew's Correlation Coefficient; AUC = Area Under Curve; BA = Balanced Accuracy.

Supplementary Table S12

Participant-Level Mean Classification Performance across 25 Iterations for the Five-Fold Cross-Validation Within-Participant Approach

Performance Metrics	on-task vs off-task	internal vs external	freely moving vs not	stuck vs not	goal-oriented vs not	self-oriented vs not	other-oriented vs not
Participant 1							
MCC	0.32 (0.02)	0.27 (0.02)	0.31 (0.02)	0.18 (0.02)	0.20 (0.03)	0.33 (0.02)	0.20 (0.03)
AUC	0.74 (0.02)	0.70 (0.01)	0.70 (0.01)	0.64 (0.01)	0.65 (0.02)	0.73 (0.01	0.67 (0.02)
BA	0.65 (0.01)	0.63 (0.01)	0.65 (0.01)	0.57 (0.02)	0.59 (0.01)	0.66 (0.01)	0.60 (0.02)
Participant 2							
MCC		0.78 (0.01)			0.75 (0.02)		0.71 (0.02)
AUC		0.95 (0.00)			0.97 (0.01)		0.97 (0.00)
BA		0.89 (0.01)			0.87 (0.01)		0.85 (0.02)
Participant 3							
MCC	0.18 (0.02)	0.22 (0.02)	0.23 (0.03)	0.28 (0.01)	0.13 (0.03)	0.51 (0.01)	0.14 (0.02)
AUC	0.62 (0.02)	0.71 (0.02)	0.66 (0.03)	0.71 (0.01)	0.59 (0.02)	0.83 (0.01)	0.58 (0.02)
BA	0.57 (0.01)	0.60 (0.02)	0.60 (0.01)	0.62 (0.01)	0.54 (0.01)	0.75 (0.01)	0.56 (0.01)
Participant 4							
MCC	0.05 (0.03)		0.10 (0.03)	0.10 (0.02)	0.61 (0.02)	0.06 (0.02)	0.55 (0.01)
AUC	0.69 (0.03)		0.74 (0.02)	0.68 (0.02)	0.85 (0.01)	0.61 (0.02)	0.84 (0.01)
BA	0.52 (0.01)		0.55 (0.02)	0.54 (0.01)	0.79 (0.01)	0.52 (0.01)	0.76 (0.01)
Participant 5							
MCC	0.56 (0.02)	0.38 (0.03)	0.35 (0.02)	0.41 (0.01)	0.25 (0.03)	0.70 (0.01)	0.55 (0.02)
AUC	0.85 (0.01)	0.78 (0.03)	0.73 (0.02)	0.81 (0.00)	0.72 (0.02)	0.91 (0.00)	0.86 (0.01)
BA	0.78 (0.01)	0.68 (0.02)	0.67 (0.01)	0.71 (0.01)	0.61 (0.02)	0.85 (0.01)	0.77 (0.01)
Participant 6							
MCC							0.43 (0.03)
AUC							0.90 (0.01)
BA							0.70 (0.02)
Participant 7							
MCC	0.14 (0.03)	0.06 (0.03)	0.10 (0.03)	0.12 (0.03)	0.14 (0.03)	0.11 (0.03)	
AUC	0.60 (0.02)	0.53 (0.02)	0.57 (0.02)	0.59 (0.02)	0.60 (0.02)	0.64 (0.02)	
BA	0.56 (0.02)	0.52 (0.01)	0.54 (0.01)	0.55 (0.02)	0.56 (0.01)	0.33 (0.02)	

Note. The identical procedure for computing classification performance at the group level was implemented for each participant, as long as they have sufficient datapoints for the upper and lower end of a given thought dimension. The classification performance as indexed by three performance metrics are reported for each participant (across rows) and thought dimension (across columns). Each value represents the mean (and standard deviation) of classification performance across all 25 iterations. MCC = Matthew's Correlation Coefficient; AUC = Area Under Curve; BA = Balanced Accuracy.

Supplementary Table S13

Participant-Level Mean Classification Performance Across 25 Iterations for the Leave-One-Participant-Out Cross-Validation Approach

Performance Metrics	on-task vs off-task	internal vs external	freely moving vs not	stuck vs not	goal-oriented vs not	self-oriented vs not	other-oriented vs not
Participant 1							
MCC	0.21 (0.04)	0.20 (0.04)	0.20 (0.04)	0.07 (0.03)	0.14 (0.03)	0.18 (0.03)	0.14 (0.04)
AUC	0.64 (0.02)	0.65 (0.02)	0.62 (0.02)	0.57 (0.02)	0.60 (0.02)	0.63 (0.02)	0.61 (0.03)
BA	0.61 (0.02)	0.61 (0.02)	0.60 (0.02)	0.54 (0.02)	0.57 (0.02)	0.59 (0.01)	0.57 (0.02)
Participant 2							
MCC		0.60 (0.04)			0.65 (0.06)		0.61 (0.06)
AUC		0.88 (0.02)			0.93 (0.02)		0.94 (0.02)
BA		0.80 (0.02)			0.84 (0.04)		0.80 (0.05)
Participant 3							
MCC	0.13 (0.04)	0.17 (0.04)	0.17 (0.04)	0.22 (0.04)	0.06 (0.03)	0.39 (0.04)	0.05 (0.04)
AUC	0.58 (0.03)	0.67 (0.02)	0.61 (0.03)	0.67 (0.02)	0.55 (0.03)	0.76 (0.02)	0.55 (0.02)
BA	0.56 (0.02)	0.59 (0.02)	0.58 (0.02)	0.61 (0.02)	0.53 (0.02)	0.70 (0.02)	0.53 (0.02)
Participant 4							
MCC	0.03 (0.04)		0.12 (0.04)	0.07 (0.04)	0.46 (0.05)	0.02 (0.04)	0.40 (0.03)
AUC	0.60 (0.05)		0.67 (0.02)	0.62 (0.05)	0.82 (0.02)	0.57 (0.04)	0.76 (0.02)
BA	0.52 (0.02)		0.56 (0.02)	0.54 (0.02)	0.73 (0.03)	0.51 (0.02)	0.71 (0.02)
Participant 5							
MCC	0.41 (0.04)	0.28 (0.05)	0.24 (0.04)	0.29 (0.05)	0.18 (0.06)	0.54 (0.03)	0.41 (0.03)
AUC	0.77 (0.02)	0.72 (0.02)	0.66 (0.02)	0.75 (0.02)	0.67 (0.04)	0.84 (0.01)	0.79 (0.02)
BA	0.70 (0.02)	0.64 (0.03)	0.62 (0.02)	0.65 (0.03)	0.59 (0.03)	0.77 (0.02)	0.71 (0.02)
Participant 6							
MCC							0.26 (0.05)
AUC							0.80 (0.04)
BA							0.63 (0.03)
Participant 7							
MCC	0.05 (0.03)	0.03 (0.04)	0.07 (0.04)	0.07 (0.04)	0.11 (0.04)	0.05 (0.05)	
AUC	0.55 (0.02)	0.53 (0.02)	0.56 (0.02)	0.56 (0.03)	0.58 (0.03)	0.61 (0.04)	
BA	0.53 (0.02)	0.52 (0.02)	0.53 (0.02)	0.54 (0.02)	0.55 (0.02)	0.53 (0.03)	

Note. The identical procedure for computing classification performance at the group level was implemented for each participant, as long as they have sufficient datapoints for the upper and lower end of a given thought dimension. The classification performance as indexed by three performance metrics are reported for each participant (across rows) and thought dimension (across columns). Each value represents the mean (and standard deviation) of classification performance across all 25 iterations. MCC = Matthew's Correlation Coefficient; AUC = Area Under Curve; BA = Balanced Accuracy. These metrics for each participant represent the classification performance trained on the remaining participants and tested on that participant, who was the held out participant in this leave-one-participant-out approach.

Supplementary Figure S1

Cluster-Based Permutation Tests on Oscillatory Markers of Visual and Auditory Thought Dimensions During Self-Selected Tasks

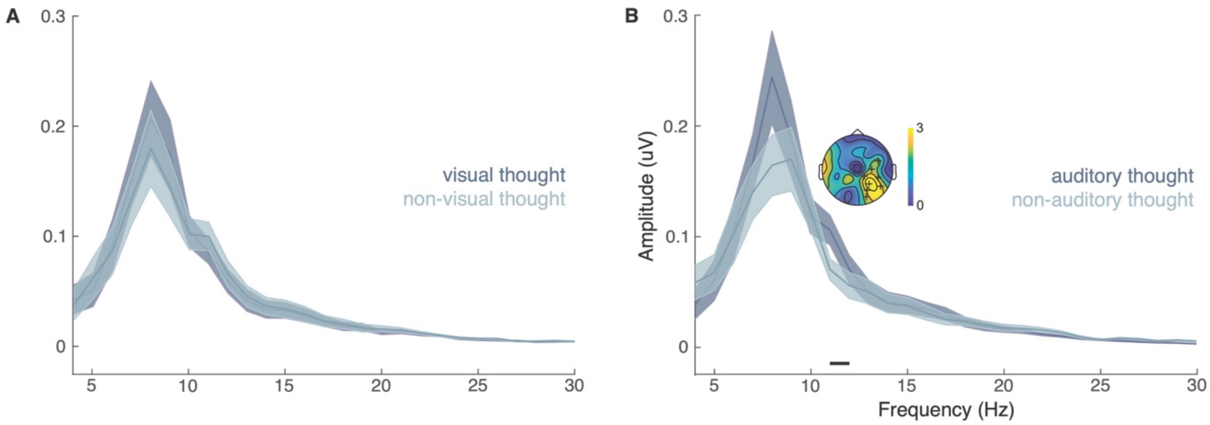

Note. A) visual thought and B) auditory thought. Each subpanel illustrates the mean EEG spectral density of the two ends of a given thought dimension across sessions and participants (with ribbons indicating the standard errors across participants). The black horizontal lines indicate timepoints of significance between the two classes of a given thought dimension based on the cluster-based permutation tests. The x and ∗ in the topoplots illustrate the significant topographic difference in spectral activity (with statistical values ranging from 0 to 3 in units of statistical scores). Warmer colors indicate greater activity during the top end relative to bottom end of a thought dimension, whereas cooler colors indicate greater activity during the bottom end compared to top of the thought dimension.

Supplementary Figure S2

Architecture of the Proposed 1 Dimensional – Convolution Neural Network used for Classification of Thought Dimensions

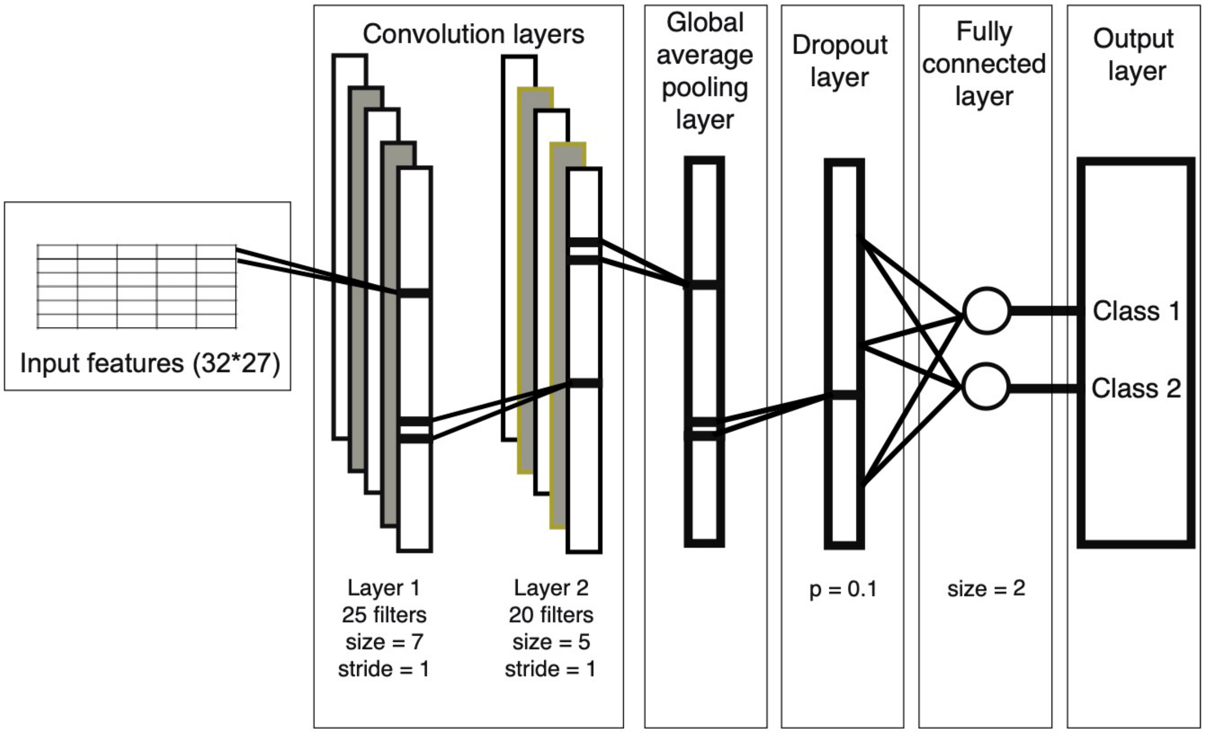

Note. The input features (32 channels x 27 frequencies) for each trial were first fed into one convolution layer in the shape of 7 kernel size * 25 filters with a stride value of 1. Next, we used one more convolution layer in the shape of 5 kernel size * 20 filters with a stride value of 1. Each convolution layer was followed by the ReLU layer for non-linear activation. Feature map outputs from the ReLU layer were then entered into a global average pooling layer. Following this is a dropout layer with a probability of 0.1. Finally, one fully connected layer with a size of 2 followed by a classification layer with the SoftMax function was implemented to classify the EEG signals into either class of a given thought dimension.

Supplementary Figure S3

Cluster-based permutation tests on oscillatory markers of thought dimensions during rest.

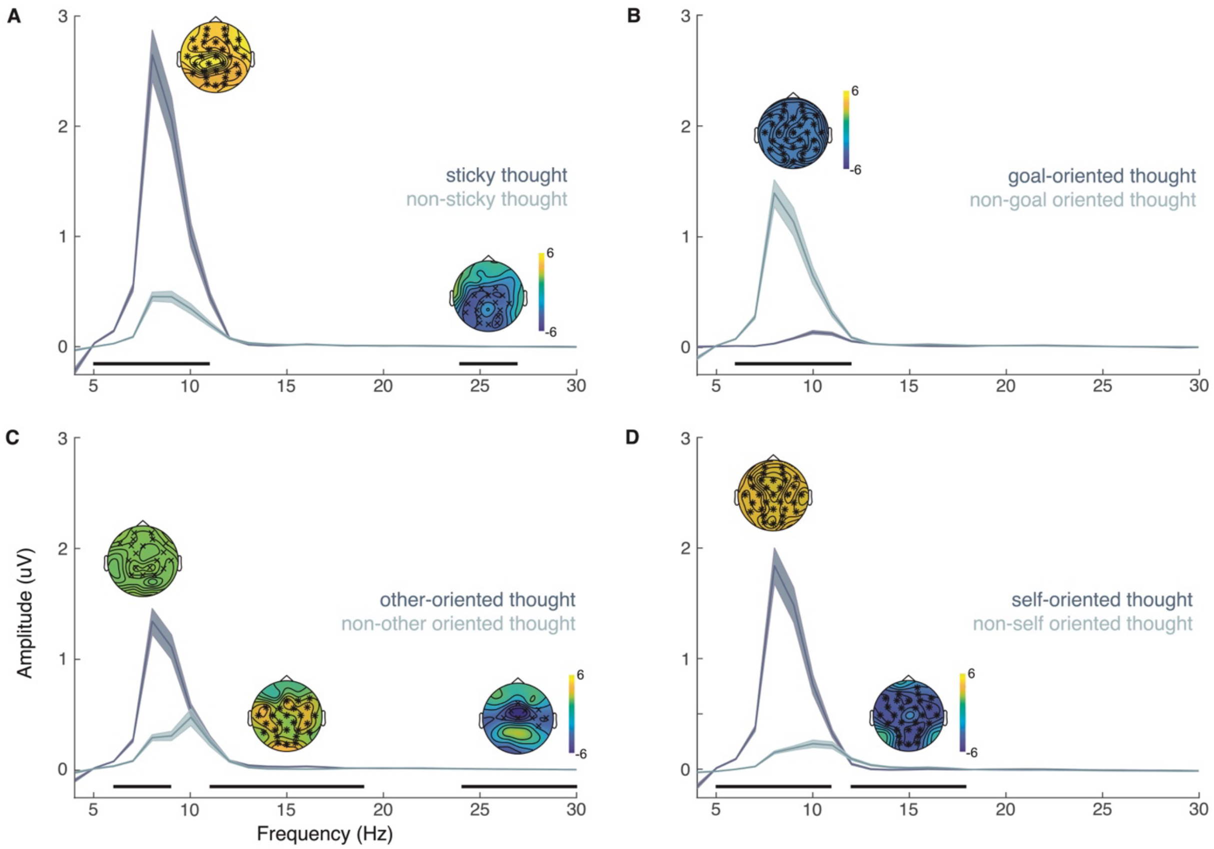

Note: (A) sticky thought, (B) goal-oriented thought, (C) other-oriented thought, and (D) self-oriented thought. Thought dimensions in which there was an absence of data on either end of the dimension across sessions for a given participant were excluded from these analyses. Each subpanel illustrates the mean EEG spectral density of the two ends of a given thought dimension across sessions and participants (with ribbons indicating the standard errors across participants). The black horizontal lines indicate timepoints of significance between the two classes of a given thought dimension based on the cluster- based permutation tests. The x and * in the topoplots illustrate the significant topographic difference in spectral activity (with statistical t-values ranging from -6 to 6 for all thought dimensions). Warmer colors indicate greater activity during the upper end relative to lower end of a thought dimension, whereas cooler colors indicate greater activity during the lower end than during upper end of the thought dimension.
